# A fully automated explainable predictive model for diagnosing pre-capillary and post-capillary pulmonary hypertension on routine unenhanced CT: results from the ASPIRE registry

**DOI:** 10.1093/ehjdh/ztaf124

**Published:** 2025-10-27

**Authors:** Turki Nasser Alnasser, Alireza Hokmabadi, Elliot W Checkley, Michael J Sharkey, Lojain F Abdulaal, Khalid S Alghamdi, Pankaj Garg, Ahmed Maiter, Krit Dwivedi, Mahan Salehi, Jonathan Taylor, Peter Metherall, Georgia A Hyde, Ze Ming Goh, David G Kiely, Samer Alabed, Andrew J Swift

**Affiliations:** School of Medicine and Population Health, The University of Sheffield, Sheffield S10 2TN, United Kingdom; Radiological Sciences Department, College of Applied Medical Science, King Saud bin Abdulaziz University for Health Science, Riyadh 11426, Saudi Arabia; King Abdullah International Medical Research Centre (KAIMRC), National Guard Health Affairs, Riyadh 11426, Saudi Arabia; School of Medicine and Population Health, The University of Sheffield, Sheffield S10 2TN, United Kingdom; Insigneo Institute, Faculty of Engineering, The University of Sheffield, Sheffield, United Kingdom; Academic Unit of Clinical Radiology, University of Sheffield, Sheffield, United Kingdom; School of Medicine and Population Health, The University of Sheffield, Sheffield S10 2TN, United Kingdom; 3D Imaging Lab, Sheffield Teaching Hospitals NHSFT, Sheffield, United Kingdom; School of Medicine and Population Health, The University of Sheffield, Sheffield S10 2TN, United Kingdom; School of Medicine and Population Health, The University of Sheffield, Sheffield S10 2TN, United Kingdom; Norwich Medical School, Faculty of Medicine and Health Sciences, University of East Anglia, Norwich, United Kingdom; School of Medicine and Population Health, The University of Sheffield, Sheffield S10 2TN, United Kingdom; Department of Clinical Radiology, Sheffield Teaching Hospitals, Sheffield, United Kingdom; School of Medicine and Population Health, The University of Sheffield, Sheffield S10 2TN, United Kingdom; Department of Clinical Radiology, Sheffield Teaching Hospitals, Sheffield, United Kingdom; School of Medicine and Population Health, The University of Sheffield, Sheffield S10 2TN, United Kingdom; Department of Clinical Radiology, Sheffield Teaching Hospitals, Sheffield, United Kingdom; 3D Imaging Lab, Sheffield Teaching Hospitals NHSFT, Sheffield, United Kingdom; 3D Imaging Lab, Sheffield Teaching Hospitals NHSFT, Sheffield, United Kingdom; Department of Clinical Radiology, Sheffield Teaching Hospitals, Sheffield, United Kingdom; School of Medicine and Population Health, The University of Sheffield, Sheffield S10 2TN, United Kingdom; School of Medicine and Population Health, The University of Sheffield, Sheffield S10 2TN, United Kingdom; Insigneo Institute, Faculty of Engineering, The University of Sheffield, Sheffield, United Kingdom; Sheffield Pulmonary Vascular Disease Unit, Sheffield Teaching Hospitals NHS Trust, Sheffield, United Kingdom; National Institute for Health and Care Research, Sheffield Biomedical Research Centre, Sheffield, United Kingdom; School of Medicine and Population Health, The University of Sheffield, Sheffield S10 2TN, United Kingdom; Insigneo Institute, Faculty of Engineering, The University of Sheffield, Sheffield, United Kingdom; Department of Clinical Radiology, Sheffield Teaching Hospitals, Sheffield, United Kingdom; National Institute for Health and Care Research, Sheffield Biomedical Research Centre, Sheffield, United Kingdom; School of Medicine and Population Health, The University of Sheffield, Sheffield S10 2TN, United Kingdom; Insigneo Institute, Faculty of Engineering, The University of Sheffield, Sheffield, United Kingdom; Department of Clinical Radiology, Sheffield Teaching Hospitals, Sheffield, United Kingdom; National Institute for Health and Care Research, Sheffield Biomedical Research Centre, Sheffield, United Kingdom; Sheffield Pulmonary Vascular Disease Unit, Sheffield Teaching Hospitals NHS Trust, Sheffield, United Kingdom

**Keywords:** Unenhanced CT, Segmentation, Deep learning, Cardiac, Pulmonary hypertension, Left Heart Disease

## Abstract

**Aims:**

Unenhanced chest CT is frequently used to assess lung malignancy and parenchymal disease. Harnessing CT data to quantify cardiac and vascular structures has the potential to improve the diagnosis of heart failure and pulmonary hypertension (PH). This study aims to develop a deep learning model to segment and analyse cardiothoracic structures from unenhanced CT images to diagnose PH, pre-capillary PH and PH associated with left heart disease (LHD).

**Methods and results:**

A twelve-structure cardiothoracic segmentation model was developed using an institutional cohort (*n* = 55, 35/9/11 training/validation/testing). Model performance was evaluated using Dice similarity coefficients (DSC). Volumetric measurements were compared to manual values using intra-class correlation (ICC) and visually assessed by four observers using an external cohort (*n* = 50, from 26 hospitals). Univariable and multivariable regression analyses were performed using a cohort of 368 patients (254/114 training/testing). Receiver-operating characteristic curves were plotted and the area under the curves (AUC) with confidence intervals (CI) were calculated. The model yielded a DSC segmentation performance of ≥0.87 for 9/12 segmented structures and ICC > 0.95 for 10/12 structures. Most of the segmented structures scored as excellent in the external cohort visual assessment. Diagnostic accuracy for predicting PH was high [AUC = 0.88 (CI: 0.80–0.96), sensitivity = 70%, specificity = 100%], including pre-capillary PH [AUC = 0.84 (CI: 0.74–0.94), sensitivity = 72%, specificity = 94%] and PH-LHD [AUC = 0.86 (CI: 0.79–0.93), sensitivity = 94%, specificity = 63%].

**Conclusion:**

A fully automated model for multi-structure cardiothoracic segmentation on unenhanced CT is achievable. The model can predict PH and identify patients with pre-capillary PH and PH-LHD with promising performance.

## Graphical Abstract

**Graphical Abstract:**
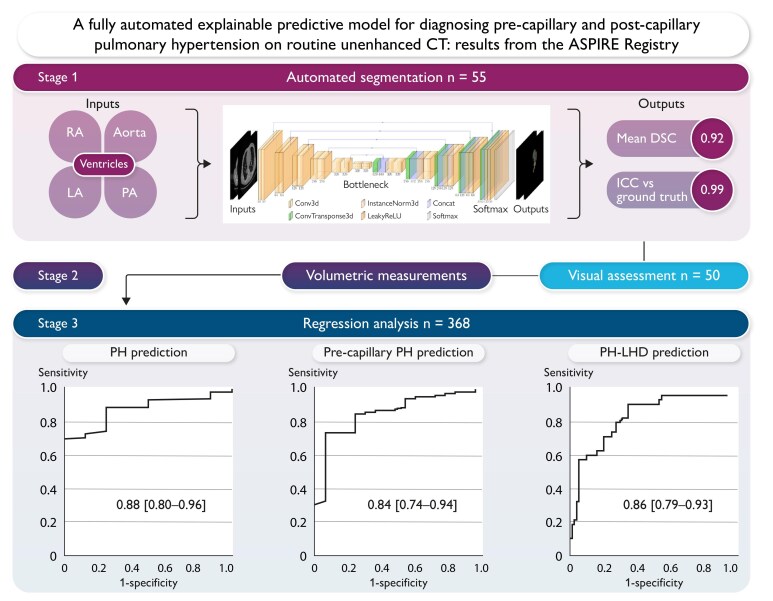

## Introduction

Routine unenhanced chest computed tomography (CT) scans performed to assess for suspected lung pathology such as emphysema, interstitial lung disease (ILD) or lung cancer provide a valuable opportunity to diagnose pulmonary hypertension (PH) and cardiac disease.^1–5^ Relying solely on visual assessment and qualitative reporting can lead to missed diagnoses with abnormalities such as pulmonary artery (PA) dilatation, atrial and ventricular and tracheal wall thickening often not appreciated.^4,6–8^ Manual measurements of these structures are both time-consuming and prone to subjective interpretation.^4,6,9^ Nonetheless, manual measurements have proven highly accurate in the detection of PA dilatation in PH^4,7^ and have prognostic value.^8,10^

Recent advances in artificial intelligence (AI) have demonstrated that automated segmentation of cardiothoracic structures can achieve high accuracy and repeatability across various imaging modalities including CT, magnetic resonance imaging (MRI), and ultrasound.^11^ This has been highlighted at the recent 7th World Symposium on PH as having potential value in the diagnosis of PH.^12^ Most studies of automated segmentation on CT have focused on contrast-enhanced acquisitions due to improved tissue differentiation.^13^ However, unenhanced acquisitions are more commonly performed in patients with suspected lung disease.^2–4^ Additionally, an unenhanced acquisition may be the only option for patients with a contrast agent allergy or renal impairment.^14^ There is overlap in the signs and symptoms with which patients with lung and cardiac disorders present, and many patients undergoing unenhanced CT for suspected lung disease have concomitant cardiac disease. As such, the ability to detect and quantify cardiac abnormalities on unenhanced CT is inherently appealing, potentially enabling earlier diagnosis.^2–4^ However, the accurate differentiation and assessment of cardiac structures in the absence of contrast is challenging and, as a result, formal assessment of these structures is rarely performed by radiologists when reporting unenhanced CT scans.^4,6^ The diagnosis of PH and its subgroups remain challenging, yet comprehensive classification is fundamental for appropriate therapeutic decision-making in accordance with the European Society of Cardiology (ESC)/European Respiratory Society (ERS) guidelines.^2^

The primary aim of this study is not to develop a new segmentation model, but rather to apply a segmentation framework to delineate major cardiothoracic structures in patients with suspected PH from unenhanced CT images.

The study aimed to:

- Assess the correlation between manual and automated volumetric measurements.
- Evaluate the diagnostic accuracy to predict PH, including pre-capillary PH (*e.g.* pulmonary arterial hypertension (PAH) and post-capillary PH (*i.e.* PH-left heart disease (LHD).
- Compare this model to traditional metrics such as mean pulmonary artery diameter (MPA) and the ratio of the PA to the diameter of the ascending aorta (AAo) (MPA/AAo).
- Validate the segmentation accuracy in an external cohort from hospitals across England and Wales.

## Materials and methods

### Ethical approval

Ethical approval for this imaging analysis study was granted and written informed consent was waived by our institutional review board [Assessing the Spectrum of Pulmonary Hypertension Identified at a Referral Centre (ASPIRE), ref: c06/Q2308/8].

### Study population

Patients were identified from the ASPIRE Registry between 2008 and 2017. Patients in the ASPIRE registry undergo systematic assessment at the Sheffield Pulmonary Vascular Disease Unit, including lung function, exercise testing, multimodality imaging and right heart catheterisation (RHC) as previously described.^15^ The registry included consecutive patients who were suspected of having PH and had not received any prior therapy. Patients were excluded if they had received such therapy before referral or if PH was attributable to multiple unrelated causes. In this study, patients were eligible for inclusion if they were aged ≥18 years, had a suspected diagnosis of PH, and underwent both RHC and unenhanced CT within one month of evaluation. Patients were excluded if they did not undergo both RHC and unenhanced CT within the specified timeframe and if RHC haemodynamic measurements (i.e. mean pulmonary arterial pressure (mPAP), pulmonary vascular resistance (PVR), and pulmonary artery wedge pressure (PAWP)) were not reported.

**Figure 1:**
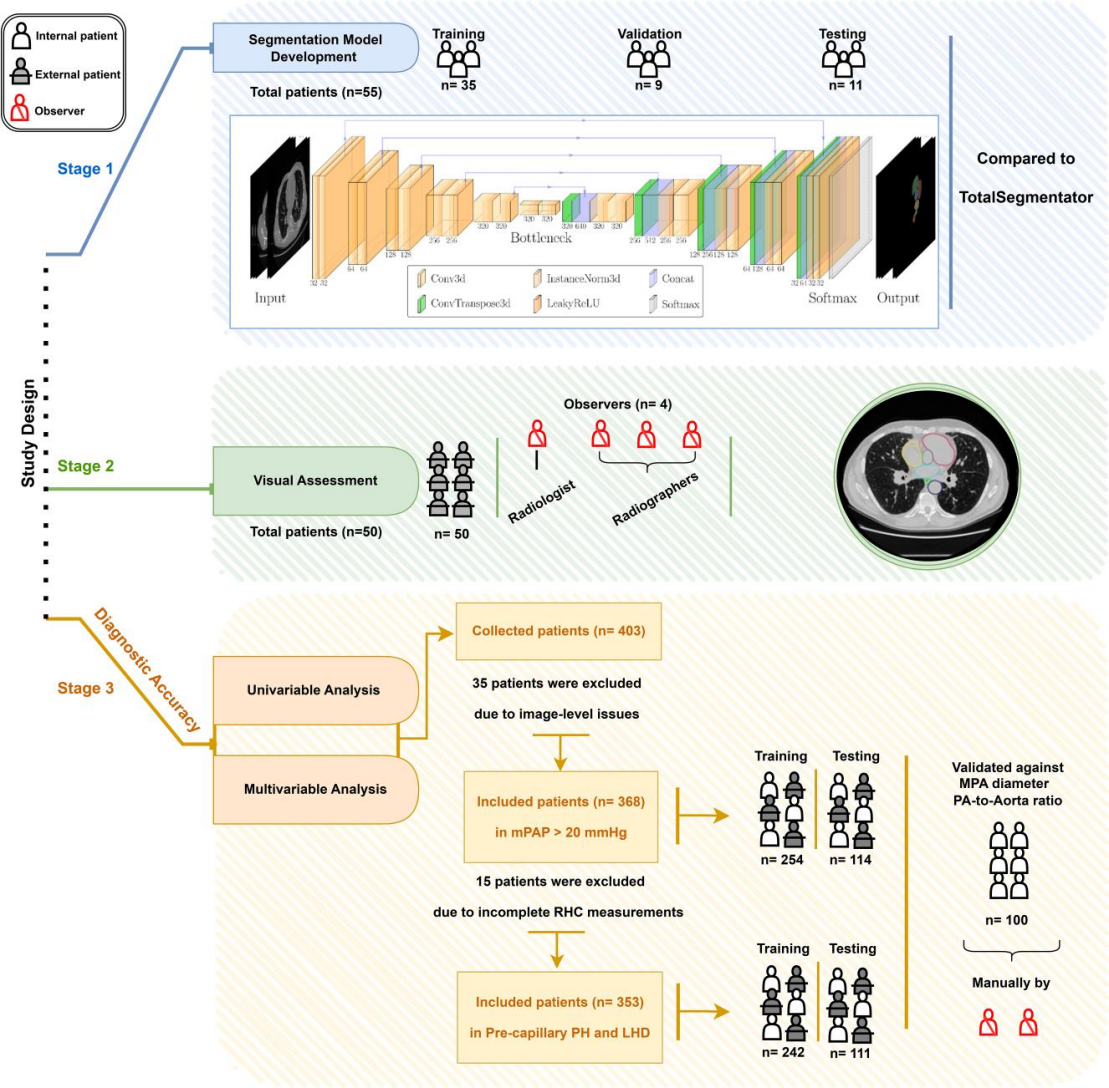
Flow chart of the study design, including (1) segmentation model development stage, (2) visual assessment stage and (3) diagnostic accuracy stage with regression analyses.

Three datasets of unenhanced chest CTs were randomly selected from the registry. A cohort of 55 unenhanced chest CTs was used to develop the deep learning segmentation model (Stage 1). To comprehensively evaluate the automated anatomical structures segmentation and volumetric measurements, an external cohort of 50 patients from 26 different hospitals across England and Wales was used in testing to visually assess the volumetric measurements (Stage 2). A dataset of 368 chest CTs was used for a regression model to predict PH (defined as mPAP > 20 mmHg) using a 70/30 training/testing split. Regression analyses were performed to generate a diagnostic model using a cohort of 353 patients (70/30 training/testing split) for the ESC/ERS PH haemodynamic subgroups, including pre-capillary PH (defined as a mPAP > 20 mmHg and PVR > 2 wood unit) and PH-LHD (defined as a mPAP > 20 mmHg and PAWP > 15 mmHg)^2^ (Stage 3). The diagnostic accuracy was preliminarily compared against manual measurements of the MPA diameter and ratio of the MPA/AAo using a cohort of 100 patients. The selected cohorts included patients with various pathology and images acquired by multi-vendor scanners (*i.e.* GE HealthCare, Siemens, and Canon) to comprehensively train the model and obtain the highest segmentation quality and diagnostic accuracy (*Figure 1*).

### CT acquisition

Unenhanced CT scans were collected from review of the ASPIRE registry database. Scans were acquired using multidetector scanners (GE MEDICAL SYSTEMS, Siemens, and Canon) with standard acquisition helical mode parameters: pitch 1, tube current 100–500 milliampere and 120 kilovolts. Patients were included if the slice thickness was 5 mm or less with a field-of-view of 400 × 400 mm and an acquisition matrix of 512 × 512.

### Image analysis and manual annotations

Images in the Digital Imaging and Communication in Medicine (DICOM) format were transferred to Medical Image Merge (MIM) software, version [7.3.3], [MIM software, Cleveland, Ohio] for manual annotation of the cardiothoracic structures. This included the ventricles as a single structure, right and left atria, ascending aorta, descending aorta, pulmonary artery (PA), mediastinal fat, oesophagus, trachea and airways, superior vena cava (SVC) and inferior vena cava (IVC). The selected anatomical structures were divided between two annotators. A cardiothoracic radiologist with 13 years of experience (AJS) labelled seven structures, including the right and left ventricles, right and left atria, ascending aorta, descending aorta and PA. A radiographer with five years of experience (TNA) labelled five structures, including the mediastinal fat, oesophagus, trachea and airways, SVC and IVC. The overall findings and annotations were checked by another cardiothoracic radiologist with seven years of experience (SA) and two senior AI research scientists (MS and AH). Manual annotation examples are provided in Supplementary material online, *Figures S1*–S10.

### Technical development of the segmentation model

For model development, 80% of the dataset (*n* = 44) was allocated for training purposes, reserving the remaining 20% (*n* = 11) for testing. To increase the validity of the training process, a five-fold cross-validation strategy was implemented within the training set. This resulted in 35 cases being utilised for training in each fold, with an additional nine cases set aside for validation. From previous experience in the fully automatic cardiac chambers and great vessel segmentation model on computed tomography pulmonary angiography (CTPA),^6^ 30–40 cases were required for training the model to achieve a good Dice similarity coefficients (DSC) score (≥ 0.90) for most of the structures (*Figure 1* and *Table 1*).

**Table 1:** Demographic information of the patient population

		Stage 1	Stage 2	Stage 3				
		Internal cohort (*n* = 55)	External cohort (*n* = 50)	Diagnostic accuracy cohort—Regression analyses				
		Model development	Visual assessment	PH prediction (*n* = 368)		PH subgroups prediction (*n* = 353)		MPA/AAo
				Train (*n* = 254)	Test (*n* = 114)	Train (*n* = 242)	Test (*n* = 111)	(*n* = 100)
**Gender**	Male	27	18	78	55	93	30	50
	Female	28	32	176	59	149	81	50
**Age (years** **±** **SD)**		63 ± 15	64 ± 12	66 ± 12	67 ± 12	67 ± 12	65 ± 12	64 ± 13
**Scan dates**		2008–2017	2008–2017	2008–2017	2008–2017	2008–2017	2008–2017	2008–2017
**Scanner manufacturers**	GE HealthCare	55	42	218	98	206	95	100
	Siemens	0	3	4	2	5	1	0
	Canon	0	5	32	14	31	15	0
**Ethnicity**	White	41	42	205	95	201	88	83
	Non-White	5	6	28	11	25	12	17
	Not stated	9	2	21	8	16	11	0
**Diagnosis**	Lung disease	24	15	60	38	66	28	18
	PAH	12	11	82	30	75	35	14
	No PH	10	4	26	9	26	9	35
	Left heart disease	5	11	66	23	55	31	18
	CTEPH	3	5	12	9	12	4	15
	PVOD	1	0	0	0	0	0	0
	PH unclear/multifactorial	0	4	6	4	6	3	0
	Borderline PH	0	0	2	1	2	1	0
**Pulmonary artery haemodynamics**	mPAP mean ± SD (mmHg)	41.2 ± 12.7	41.0 ± 12.4	41.3 ± 14.1	41.7 ± 13.5	42.1 ± 14.3	39.5 ± 14.0	38.9 ± 17.0
	PVR mean ± SD (dynes·sec·cm⁻⁵)	491.3 ± 356.2	482.3 ± 346.1	529.2 ± 404.5	564.5 ± 411.7	540.6 ± 411.9	503.6 ± 388.1	509.4 ± 447.2
	PAWP mean ± SD (mmHg)	13.5 ± 5.7	13.9 ± 5.0	13.6 ± 6.6	12.7 ± 5.4	14.2 ± 7.1	12.4 ± 5.3	12.0 ± 6.5

PAH, Pulmonary arterial hypertension; CTEPH, chronic thromboembolic pulmonary hypertension; PVOD, Pulmonary veno-occlusive disease; mPAP, mean pulmonary arterial pressure; PVR, pulmonary vascular resistance; PAWP, pulmonary artery wedge pressure; MPA, mean pulmonary artery; AAo, ascending aorta.

A deep learning model for multi-structure cardiothoracic segmentation was developed using the nnU-Net framework,^16^ which provides a self-configuring baseline for medical image segmentation. While nnU-Net served as a starting point, achieving optimal performance required careful dataset preparation and tuning of training parameters. The model was further adapted to our dataset and clinical task through customised preprocessing, resampling, and training strategies.

The model was trained for 400 epochs with a batch size of 2, using a hybrid loss function combining Dice and Cross-Entropy terms to balance segmentation across structures of varying sizes. Preprocessing involved cropping to exclude zero-value background regions, intensity clipping to the 0.5–99.5%ile range, and standardisation based on foreground voxel statistics. All scans were then resampled to a uniform voxel spacing of (0.8, 0.7, 0.7) mm to ensure spatial consistency. This spacing reflected the most common resolutions in the dataset, reducing unnecessary interpolation and preserving anatomical detail. Additionally, patch-based training removed the need to standardise volume size or voxel spacing, allowing flexibility in choosing values that best matched the data. To mitigate overfitting, given the limited dataset, we applied extensive data augmentation. Dice similarity coefficients were consistent across cross-validation folds, and validation losses remained stable across epochs (see Supplementary material online, *Figure S11*). High visual assessment scores in an external multicentre cohort further support the model’s robustness and generalisability.

To address the inherent class imbalance caused by varying anatomical structure sizes, the training process employed adaptive patch sampling to increase the presence of smaller structures (e.g. IVC, SVC) and used foreground oversampling to ensure more balanced input during training. The hybrid loss function helped further mitigate imbalance, while five-fold cross-validation improved generalisation and reduced bias toward larger structures.

**Figure 2:**
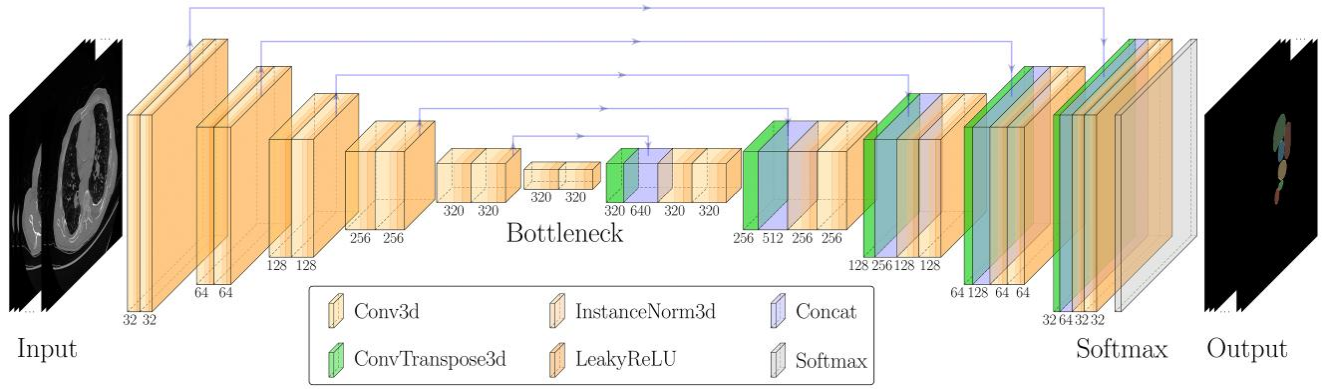
Model structure with layer details.

The network architecture was based on a modified 3D U-Net design,^17^ incorporating 3D convolutions, Instance Normalisation, and LeakyReLU activations. Downsampling and upsampling were implemented using strided and transposed convolutions, respectively. The model structure and layer configuration are illustrated in *Figure 2*.

This comprehensive approach ensures the robustness and adaptability of the model for unenhanced CT image segmentation. For external comparison, available labels from different TotalSegmentator^18^ tasks were combined to enable comparison of overlapping structures, despite differences in certain structure definitions (e.g. combined aortic segments) and the absence of some classes. The trained model will be made available upon reasonable request for research purposes. Requests should be directed to the corresponding author.

### Volumetric measurements visual assessment

To comprehensively evaluate the segmentation and volumetric measurements of the anatomical structures, the external cohort’s images (*n* = 50) were segmented by the model and the volumes of the anatomical structures were assessed independently by four reporters following a quality assessment scale where ‘excellent’ indicates highly reliable segmentation; ‘minor error’ denotes errors present but not affecting measurements; and ‘significant error’ refers to errors considered by the observer to affect the measurements (see Supplementary material online, *Table S1*). The reporters were a cardiothoracic radiologist with 13 years of experience (AJS), a cross-sectional imaging radiographer with five years of experience (TNA), a cross-sectional imaging radiographer with six years of experience (KA), and a cross-sectional imaging radiographer with 10 years of experience (LA).

### Exploratory comparison with MPA and AAo diameters

The diagnostic accuracy of the volumetric measurements in predicting PH was compared with the MPA diameter and MPA/AAo ratio. For comparison, a threshold of >29 mm in MPA diameter was applied as previously reported in^2^; this exploratory comparison may not directly reflect our cohort, and accuracy is reported for information; no direct performance comparison should be inferred. They were measured by two observers (AJS and TNA). The observers were blinded to the clinical and imaging data, as well as to each other’s measurements and AI results.

### Statistical analysis

Segmentation quality was assessed using two types of evaluation metrics, including region-based (*i.e.* DSC) and surface distance (*i.e.* Hausdorff distance) indicators following previous recommendations.^13,19^ F1 score values were calculated based on recall and precision.^20^ The volumes of anatomical structures were measured and compared with the manual measurements by calculating the difference and correlation using intra-class correlation (ICC) following the recommendations of.^21^ ICCs were calculated using the SPSS statistical package (version 29, SPSS Inc, Chicago, IL) based on a single-rater and absolute-agreement. Bland-Altman plots were created using GraphPad Prism version 10.0.0 (Boston, Massachusetts USA). Free Marginal Fleiss’ Kappa was used as the inter-rater agreement metric in the visual assessment, as it accounts for raters not assessing all categories, reducing bias and addressing variations in category prevalence.^22^ Independent T-test was used to compare patients with PH and patients without PH using manual measurements of the MPA/AAo ratio.

The regression model was generated using SPSS modeler (version 18.3) and SPSS statistics (version 29.0.1.0). Univariable analyses were performed to ascertain predictor importance for ESC/ERS PH haemodynamic definitions. Multivariable backward logistic regression analyses were performed to generate diagnostic models using a 70/30 training/testing split for the aforementioned ESC/ERS PH haemodynamic definitions. This split ratio was chosen in line with the recommendations.^23^ Univariable and multivariable analyses were conducted using the volumetric measurements of the segmented structures. The reference standard was determined using RHC measurements taken within 30 days or less, with the majority obtained on the same day (85%). Receiver-operating characteristic (ROC) curves were plotted and the area under the curve (AUC) were calculated for both univariable and multivariable models. Positive Predictive Value (PPV), Negative Predictive Value (NPV), sensitivity, and specificity were calculated using the maximal Youden index to determine the classification cut-off for multivariable models. To ensure transparency and consistency in the analysis, major anatomical structures for which our segmentation model achieved excellent performance (as indicated by high DSC and low HD scores) were included as inputs for the multivariable analysis predictions.

## Results

### Patient characteristics

The demographics of the included patients are summarised in *Table 1*. The segmentation model development cohort (*n* = 55) includes approximately equal numbers of males and females with a mean age of 63 ± 15 years. Of those, the majority of patients had pre-capillary PH with, 12 patients diagnosed with PAH and one pulmonary veno-occlusive disease (PVOD) (Group 1), 24 with PH in association with lung disease (Group 3), three with chronic thromboembolic pulmonary hypertension (CTEPH) (Group 4), five with post-capillary PH, PH-LHD (Group 2), and 10 patients with non-specific shortness of breath (non-PH). The visual assessment validation external cohort (*n* = 50) included 18 males and 32 females with a mean age of 64 ± 12 years. The majority of the external cohort were diagnosed with lung disease (*n* = 15), PAH (*n* = 11), LHD (*n* = 11) and 4 patients with non-specific shortness of breath (non-PH). The PH diagnostic accuracy cohort (*n* = 368) includes 133 male and 235 female patients with a mean age of 66 ± 12 while the pre-capillary PH and PH-LHD diagnostic accuracy cohort (*n* = 353) includes 123 male and 230 female patients. The MPA/AAo cohort (*n* = 100) includes equal numbers of males and females with a mean age of 64 ± 13 years and 65 patients with PH. The majority of the patients were scanned with GE HealthCare (87.9%), followed by Canon (10.5%) and Siemens (1.6%).

### Segmentation evaluation and performance metrics—internal cohort (stage 1)

Most of the segmented cardiothoracic structures exceeded a DSC score of ≥ 0.87. However, the mediastinal fat, IVC and oesophagus DSC scores were low in comparison to other structures. Based on a previous systematic review,^13^ a high DSC score is not the sole metric for assessing segmentation quality; Hausdorff distance was reported to comprehensively evaluate the results. The proposed model was evaluated against TotalSegmentator^18^ and demonstrated comparable performance across overlapping structures. The majority of the segmented structures showed statistically significant F1 scores. The manual volumetric measurements of the majority of these anatomical structures were correlated with the AI-measured ones and an ICC was conducted to compare the measurements. (*Table 2* and Supplementary material online, *Figure S12*). Volumes were computed by summing labelled voxels and multiplying by voxel spacing (x × y × z), followed by conversion from mm³ to mL. Full results are provided in Supplementary material online, *Tables S2*–S5.

**Table 2:** Internal cohort segmentation development evaluation metrics compared to TotalSegmentator, performance metrics, and volume measurements correlations

	Proposed model		Total segmentator		Average Performance metrics			Volume measurements		ICC
	DSC	HD_95_	DSC	HD_95_	Recall	Precision	F1 Score	Reference mean ± SD (ml)	Prediction mean ± SD (ml)	
Ventricles	0.95	1.2	0.88	4.7	0.94	0.95	0.95	382 ± 75	379 ± 67	0.99
LA	0.87	1.6	0.83	3.8	0.89	0.86	0.87	65 ± 32	68 ± 35	0.99
RA	0.90	1.7	0.85	2.5	0.89	0.91	0.90	130 ± 63	126 ± 57	0.99
AAo	0.94	0.9	0.86^a^	4.9^a^	0.94	0.95	0.94	135 ± 33	135 ± 32	0.99
DAo	0.95	1.0			0.96	0.94	0.95	126 ± 34	128 ± 31	0.99
PA	0.90	1.6	0.77	5.9	0.91	0.89	0.90	123 ± 34	125 ± 32	0.99
Oesophagus	0.79	5.7	0.71	12.8	0.73	0.86	0.79	44 ± 11	36 ± 5	0.22
Trachea and airways	0.90	2.0	0.79	114.7	0.91	0.90	0.90	57 ± 18	58 ± 18	0.98
Mediastinal fat	0.75	40.1	NA	NA	0.74	0.80	0.77	96 ± 95	88 ± 86	0.97
SVC	0.87	1.4	0.74	5.0	0.87	0.88	0.88	26 ± 7	25 ± 6	0.96
IVC	0.74	8.7	0.58	15.1	0.77	0.75	0.76	38 ± 16	38 ± 14	0.85

LA, Left atrium; RA, Right atrium; AAo, Ascending aorta; Dao, Descending aorta; PA, Pulmonary artery; SVC, Superior vena cava; IVC, Inferior vena cava; DSC, Dice similarity coefficient; HD, Hausdorff Distance; ICC, Intraclass correlation; NA, Not applicable

^a^TotalSegmentator segmented one structure (Aorta), including AAo and DAo.

### Volumetric measurements visual assessment—external cohort (stage 2)

**Figure 3:**
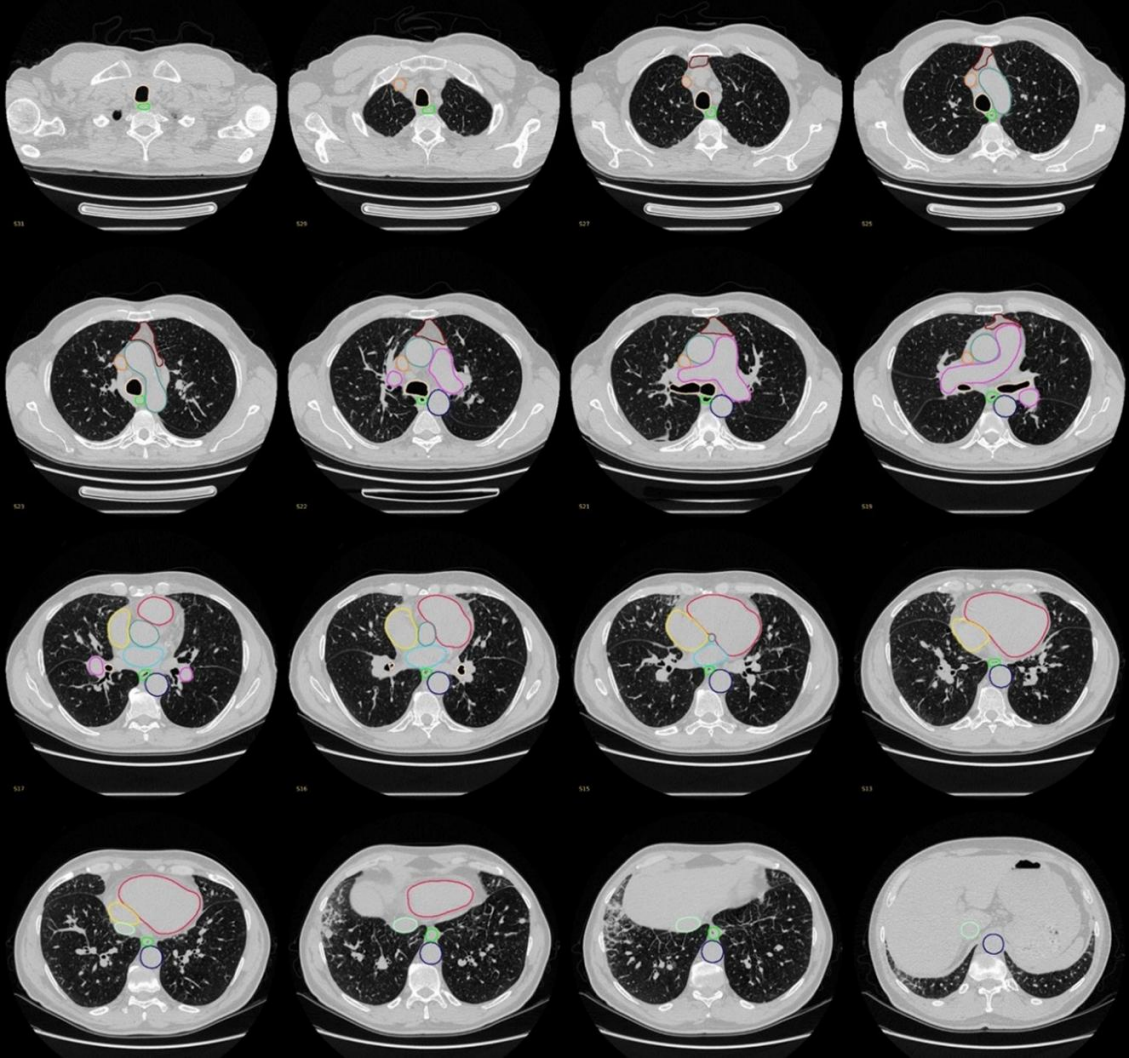
Example of a successful segmentation.

*Figure 3* shows an example of successful segmentations of our model. The visual assessment and results correlated with the aforementioned model development numerical results (*i.e.* DSC) and the majority of the segmented structures were scored as excellent. The segmentation of the oesophagus and IVC received lower scores compared to the other structures. The independent observers’ visual assessment and Kappa results are summarised in *Table 3*.

**Table 3:** Visual assessment of the volumetric measurements/segmentation using an external cohort (*n* = 50)

	Scale (%)	Ventricles	Left atrium	Right atrium	Ascending aorta	Descending aorta	Pulmonary artery	Oesophagus	Trachea and airways	Mediastinal fat	Superior vena cava	Inferior vena cava
**Reporter 1**	**Excellent**	98	98	98	100	100	98	68	96	96	100	92
	**Minor error**	0	2	0	0	0	0	12	0	2	0	6
	**Significant error**	2	0	2	0	0	2	20	4	2	0	2
**Reporter 2**	**Excellent**	96	96	96	100	100	98	78	96	90	96	74
	**Minor error**	2	4	2	0	0	2	16	2	8	4	20
	**Significant error**	2	0	2	0	0	0	6	2	2	0	6
**Reporter 3**	**Excellent**	98	98	98	82	96	100	66	94	96	98	94
	**Minor error**	0	2	0	18	4	0	14	0	4	2	2
	**Significant error**	2	0	2	0	0	0	20	6	0	0	4
**Reporter 4**	**Excellent**	96	98	98	90	92	96	68	96	90	98	90
	**Minor error**	2	2	0	10	6	4	20	2	10	0	6
	**Significant error**	2	0	2	0	2	0	12	2	0	2	4
**Average**	**Excellent**	**97%**	**97.5%**	**97.5%**	**93%**	**97%**	**98%**	**70%**	**95.5%**	**93%**	**98%**	**87.5%**
	**Minor error**	**1%**	**2.5%**	**0.5%**	**7%**	**2.5%**	**1.5%**	**15.5%**	**1%**	**6%**	**1.5%**	**8.5%**
	**Significant error**	**2%**	**0%**	**2%**	**0%**	**0.5%**	**0.5%**	**14.5%**	**3.5%**	**1%**	**0.5%**	**4%**
**Interobserver variability metrics**	**Kappa**	**0.97**	**0.93**	**0.98**	**0.79**	**0.92**	**0.95**	**0.69**	**0.96**	**0.84**	**0.94**	**0.75**
	**Agreement Percentage**	**96%**	**92%**	**98%**	**72%**	**92%**	**94%**	**68%**	**96%**	**82%**	**92%**	**72%**

### Diagnostic accuracy (stage 3)

#### Univariable analyses

**Figure 4:**
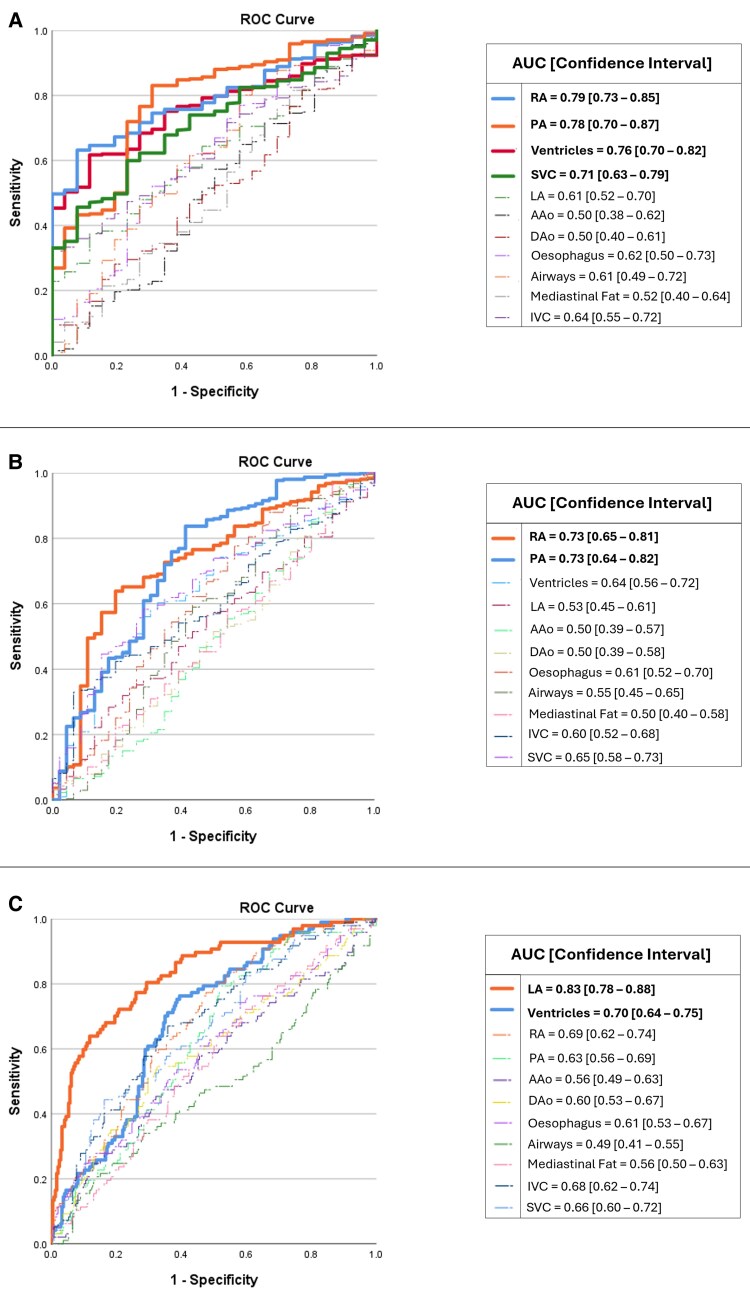
AUC and ROC univariable analysis for the prediction of *A* PH (*B*) pre-capillary PH (*C*) PH-left heart disease.

The univariable model demonstrated predictive capability for PH (mPAP > 20 mmHg) by identifying significant associations with increases in right atrium (RA), PA, ventricular and SVC volumes, yielding AUC values of 0.79, 0.78, 0.76 and 0.71 respectively (*Figure 4A*). Additionally, in patients with pre-capillary PH (mPAP > 20 mmHg and PVR > 2 wood unit), RA and PA volumes achieved AUC values of 0.73 equally (*Figure 4B*). On the other hand, the left atrium (LA) volume was the most reliable predictor (AUC = 0.83), followed by ventricles (AUC = 0.70) in predicting PH-LHD patients (*Figure 4C*).

#### Multivariable analyses

**Figure 5:**
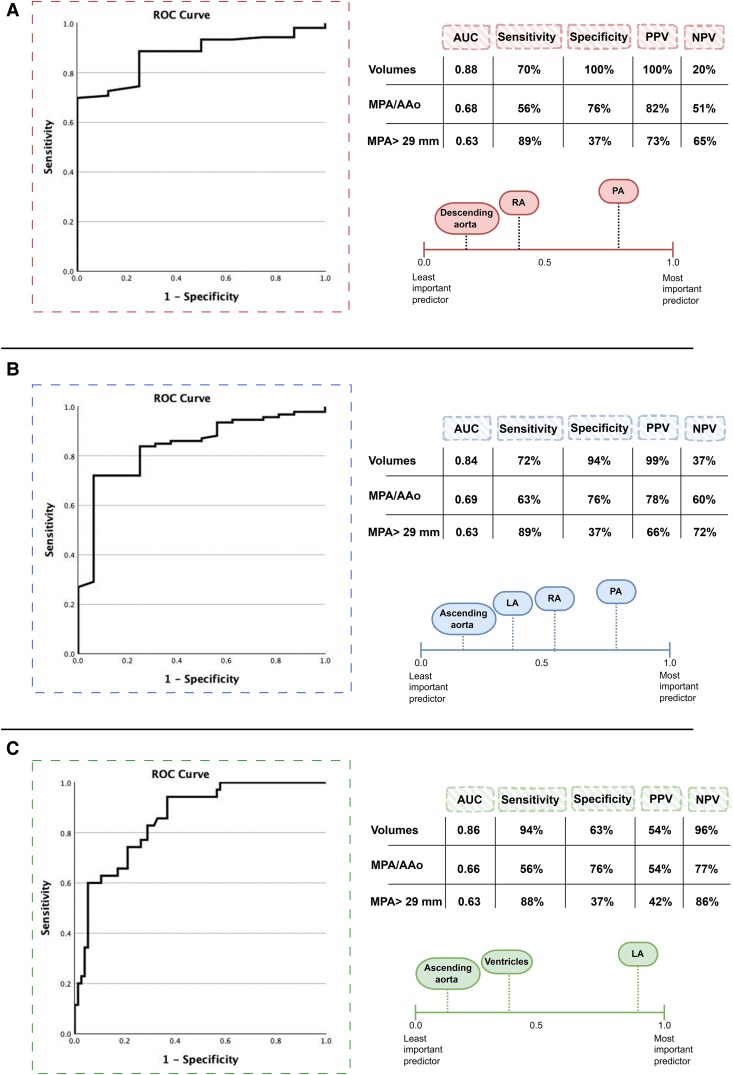
Comparison between the multivariable analysis ROC curves of AI cardiothoracic volumetric measurements and mean pulmonary artery (MPA) and ascending aorta (AAo) diameters in predicting (*A*) PH (*B*) pre-capillary PH (*C*) PH-LHD.

The cardiac chambers and the main great vessels (*i.e.* ascending aorta, descending aorta, and PA) were selected as inputs for the multivariable analysis to preserve the consistency as they showed high performance metrics. The multivariable model demonstrated robust predictive performance for PH overall, achieving an AUC of 0.88 [confidence interval (CI): 0.80–0.96] with a sensitivity of 70% and specificity of 100%, using a classification cut-off of 0.95. For pre-capillary PH, the model yielded an AUC of 0.84 (CI: 0.74–0.94) with sensitivity and specificity of 72% and 94% respectively, using a classification cut-off of 0.85. For predicting PH-LHD, the model showed an AUC of 0.86 (CI: 0.79–0.93) with 94% sensitivity and 63% specificity, using a classification cut-off of 0.13 (*Figure 5* and *Table 4*).

**Table 4:** Multivariable regression coefficients for anatomical structure volumes. The most significant predictor is indicated in bold

	Multivariable equation (volume variables)
**PH**	0.0024 * Ventricles −0.004013 * LA + 0.0145 * RA −0.007727 * Ascending aorta + 0.03705 * PA −0.02648 * Descending aorta + 0.2849
**Pre-capillary PH**	−0.004181 * Ventricles −0.01502 * LA + 0.02051 * RA −0.01535 * Ascending aorta + 0.02813 * PA −0.003064 * Descending aorta + 1.287
**PH-LHD**	0.004671 * Ventricles + 0.03636 * LA −0.003954 * RA −0.02266 * Ascending aorta + 0.001275 * PA + 0.00771 * Descending aorta −3.877

PH, Pulmonary hypertension; PH-LHD, Pulmonary hypertension associated with left heart disease; LA, Left atrium; RA, Right atrium; PA, Pulmonary artery.

#### Exploratory comparison with MPA and AAo diameters

The observers’ independent measurements of the MPA/AAo ratio were highly consistent with an ICC of 0.87. A statistically significant difference was observed between patients with PH and those without, with a *P*-value of less than 0.001 (see Supplementary material online, *Tables S6* and *S7*). The MPA/AAo ratio using a threshold > 1 was able to identify PH patients [AUC = 0.68 (CI: 0.59–0.75), sensitivity = 56%, specificity = 76%], pre-capillary PH [AUC = 0.69 (CI: 0.60–0.76), sensitivity = 63%, specificity = 76%], and PH-LHD [AUC = 0.66 (CI: 0.53–0.76), sensitivity = 56%, specificity = 76%]. The MPA diameter using a threshold > 29 mm was able to predict PH with low specificity but high sensitivity (*i.e.* 37% and 89% respectively) (*Figure 5* and Supplementary material online, *Figure S13*).

## Discussion

The proposed model was trained on unenhanced CT images for the segmentation of multi-cardiothoracic structures and prediction of PH, including pre- and post-capillary using volumetric measurements. In clinical practice the cardiac structures on unenhanced CT are not typically evaluated in detail by radiologists due to lack of visible landmarks.^4,6^ Taking the study’s limitations into account, the results of this exploratory study demonstrate that an AI segmentation model can assess the majority of the structures as well as provide volumetric measurements for diagnostic purposes, with promising performance and reasonable accuracy.

There is potential to improve diagnostic radiology through quantitative analysis in addition to standard qualitative radiological assessments, allowing features to be identified that might be otherwise overlooked.^3,6,24^ A previous study^4^ evaluated the diagnostic value of simple manual diameter and area measurements and concluded that the diagnostic accuracy was in the range of acceptable to excellent. Another group^25^ showed that quantification of cardiac structures using unenhanced CT scans plays an important role in the detection of the early cardiac changes to enable detection before clinically evident heart failure. Our findings are in agreement with those of the previous studies and this study further provides evidence of promising quality and performance in unenhanced CT segmentation by deep learning.

While various deep learning architectures have demonstrated strong performance in medical image segmentation,^11,13^ nnU-Net was utilised as a self-configuring framework to develop a model tailored to our specific clinical task. To ensure accuracy and reliability, the model was adapted through dataset preprocessing, adjustments to the training strategy, and targeted validation.

Our goal was to develop a segmentation model that remains robust across anatomical variability, scanner differences, and subtle boundaries in unenhanced CT. Design choices such as customised data augmentation and systematic validation contributed to generalisability and helped mitigate overfitting, particularly in small or low-contrast structures relevant to cardiopulmonary disease.

Although TotalSegmentator^18^ was not specifically optimised for a PH population, benchmarking our model against this validated and widely used tool offers valuable context and strengthens the technical validation.

The model achieved an excellent DSC score of 0.95 and excellent HD_95_ values (mean of 1.2) in ventricular segmentation. Moreover, it continues to yield high DSCs with LA and RA scores of 0.87 and 0.90 respectively. In relation to previously reported segmentation models,^13^ our LA and RA segmentation model demonstrated promising performance relative to contrast-enhanced models,^6,26–30^ acknowledging the cohorts are not matched. We postulate that this performance is due to utilising the network architecture to optimise hyperparameters automatically and enhance the segmentation quality by using a hybrid loss function.^16^

Different imaging features can be used to detect PH, including PA dilation, right ventricular (RV) hypertrophy and interventricular septal flattening.^31,32^ PA dilatation is one of the most common quantitative CT measurements used to predict PH, providing diagnostic and prognostic value.^3,33^ This study achieved accurate results for PA segmentation (DSC = 0.90 and HD_95_= 1.6), which is comparable to values reported in gated contrast-enhanced CT models.^26,29,34^ Despite the lack of contrast, the model yielded high DSCs in ascending and descending aorta segmentation of 0.94 and 0.95 respectively. Furthermore, the model’s volume measurements of the PA and aorta were highly correlated with the manual measurements (ICC = 0.99).

To have a comprehensive segmentation model the trachea and airways, oesophagus, SVC, IVC and mediastinal fat were also included. A Good performance in SVC segmentation was achieved, with results favourable to those of the contrast-enhanced model.^30^ Despite achieving acceptable DSC scores of the IVC and mediastinal fat (0.74 and 0.75 respectively), the model failed in delineating the borders of the structures leading to a negative volume effect, which can be seen by the HD_95_ values of 8.7 and 40.1 respectively^19,35^ (see Supplementary material online, *Figure S14* and Supplementary material online, *Table S8*). Because the model was developed using a small cohort, a larger external cohort was tested and visually assessed by four observers to validate the segmentation numerical values. Most of these structures were evaluated as highly reliable and excellent segmentation. The model showed perfect segmentation of the ventricles, LA, RA, PA and SVC in approximately 49 images out of 50 (97%—98%).

The majority of the previous PH diagnostic accuracy CT studies were focused on CTPA to assess PA size, which has been proposed to have diagnostic value in the assessment of PH.^4^ In this study, internally and externally validated and tested automated volumes of anatomical structures were used to predict the PA size and evaluate the diagnostic accuracy for PH using unenhanced CT images. The main pathological process in pre-capillary PH is raised pulmonary vascular resistance, which increases RV afterload. As a compensatory response the RA and RV dilate. Similar to how the SVC dilates as a result of elevated venous return and right-sided heart failure, the PA dilates in response to increased pressure in the pulmonary circulation.^2,36,37^ The univariable analysis results indicated sound performance and a meaningful correlation with the aforementioned main pathological process, where RA, PA and SVC were the highest importance predictors in pre-capillary PH. Moreover, the multivariable analysis indicated that the model can reliably identify non-PH patients, with a low rate of false positives in the PH cohort, although further validation with a balanced cohort is required. Conversely in PH-LHD (PAWP > 15 mmHg), the increased pressure originates from the left side of the heart due to ventricular or valvular dysfunction (*i.e.* post-capillary PH), which leads to LA dilatation.^2,4,36,37^ It was concluded by the univariable analysis that LA dilatation is an important predictor of PH-LHD with an AUC of 0.83. In addition, multivariable analysis confirmed the same results with an AUC of 0.86. The model was able to identify patients with PAWP > 15 mmHg with a sensitivity of 94%, however the specificity was low (63%). This may reflect in part that the regression model was trained and tested using a dataset with more patients with PH. The distinction between PA, RA, and SVC dilatation in pre-capillary PH vs. LA dilatation in PH-LHD provides a valuable insight into the differential diagnosis of PH subtypes. The volumetric changes of these structures can serve as important clinical predictors for PH diagnosis and prognostic assessment of heart failure in both pre-capillary and post-capillary PH patients.

## Study limitations

The model in this exploratory study was developed using a small cohort (*n* = 55) and the sample size was not determined by *a priori* power analysis. However, diagnostic utility was tested in a larger cohort of 368 patients undergoing RHC for suspected PH. In addition, the model was compared with TotalSegmentator^18^ and was tested using an external cohort (*n* = 50) with another type of evaluation (*i.e.* visual assessment by 4 observers) to enable a comprehensive evaluation. The model failed in differentiating between the trachea and oesophagus in some cases. Calcifications or metal artefacts or a dilated oesophagus can negatively affect the segmentation quality (see Supplementary material online, *Figure S14* and Supplementary material online, *Table S8*). The model achieved slightly lower segmentation performance for the IVC, oesophagus, and mediastinal fat due to their known visually anatomical variability and the challenge of delineating their borders. However, as an unenhanced CT is an important acquisition often used in patients with suspected PH, it is helpful to have an AI model that can rapidly segment the cardiothoracic structures, measure the volumes and predict the likelihood of PH. Interobserver comparison was not performed in the model development stage due to manual annotations being equally divided between two observers. However, all results were visually checked by a cardiothoracic radiologist and AI scientist. Whilst the typical use case of this tool may be in lung disease or lung cancer screening populations, this study was conducted in a suspected PH cohort of patients who had RHC, encompassing a broader population including patients with and without lung disease, left heart disease, chronic thromboembolic disease, and miscellaneous causes of PH. Given the high prevalence of PH in this cohort, diagnostic accuracy may differ in populations with lower prevalence and further validation in a larger cohort, including more balanced datasets with a greater number of non-PH patients, would be valuable to confirm the utility and generalisability of the model.

## Conclusion

Segmentation and volumetric measurements of the cardiothoracic structures have the potential to play an important role in highlighting the possibility of PH. Although unenhanced CT lacks obvious diagnostic landmarks and is ungated, it is regularly performed and qualitatively assessed in patients with unexplained breathlessness. With the increasing integration of AI models in the medical imaging analysis field, this preliminary study presents a fully automatic model for multi-cardiothoracic structure segmentation using unenhanced CT images. The AI volumetric measurements were correlated with the manual measurements based on evaluation and performance metrics as well as visual assessments. These automated measurements have the potential to aid the diagnosis and classification of PH.

## Supplementary Material

ztaf124_Supplementary_Data

## Data Availability

The data underlying this article will be shared on reasonable request to the corresponding author.
