# Supplementary material for: A fully automated explainable predictive model for diagnosing pre-capillary and post-capillary pulmonary hypertension on routine unenhanced CT: results from the ASPIRE registry
Source: Eur Heart J Digit Health. 2025 Oct 27;7(1):ztaf124. doi: 10.1093/ehjdh/ztaf124 (PMC12821070; doi:10.1093/ehjdh/ztaf124)
Supplement: ztaf124_Supplementary_Data [file ztaf124_supplementary_data.docx]

**Supplementary Materials**

**Supplemental Figures**

Right and left ventricles were annotated together because the interventricular septum cannot be seen due to contrast agents not being used within the cohort. Supplemental Figure 1 shows an example of the right and left ventricle manual segmentation.


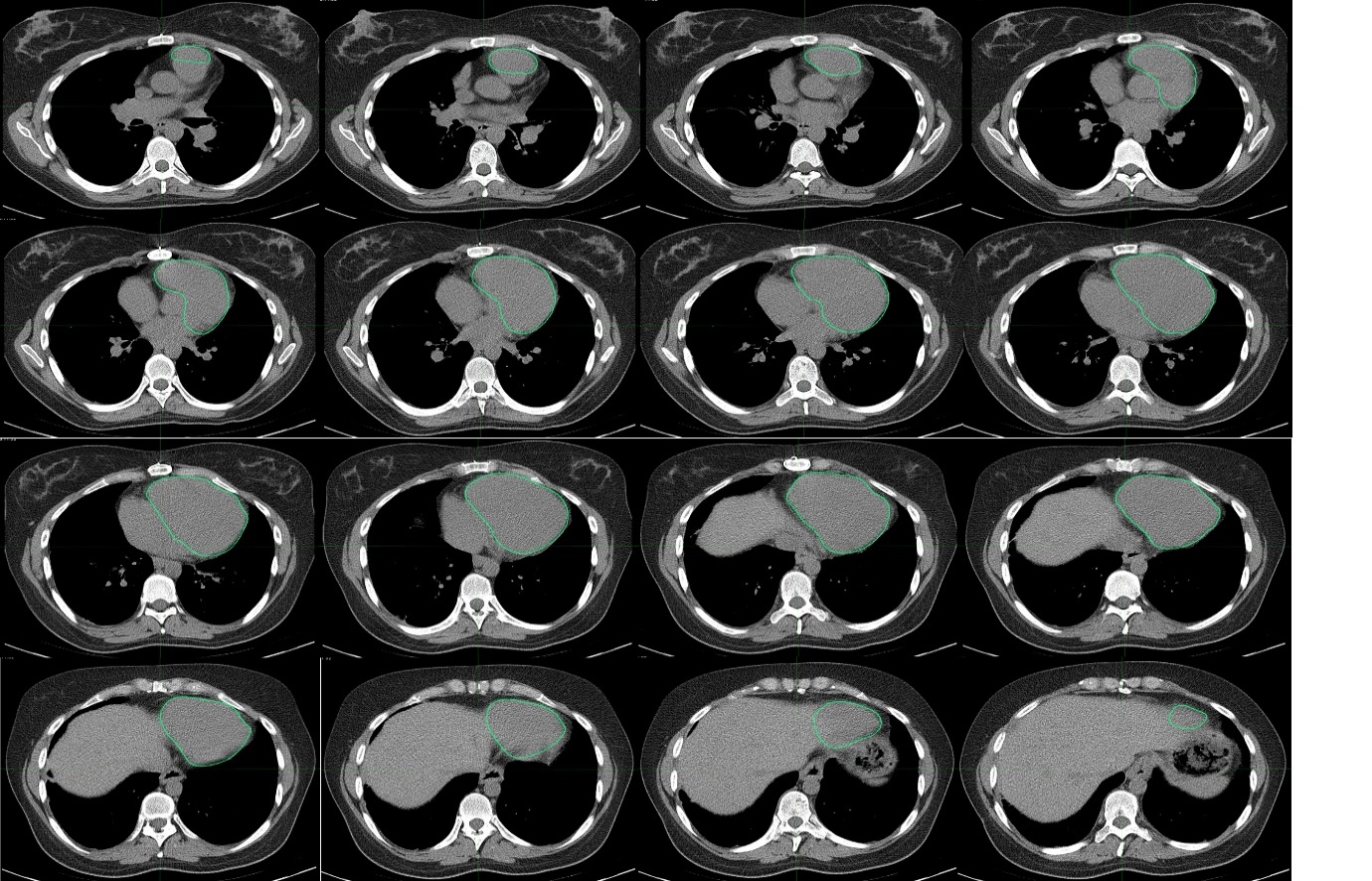


**Supplemental Figure 1.** right and left ventricle manual annotations.

RA annotation starts from the end of the SVC and ends at the start of the IVC. The right atrial appendage was excluded. Supplemental Figure 2 presents an example of the RA manual segmentation.


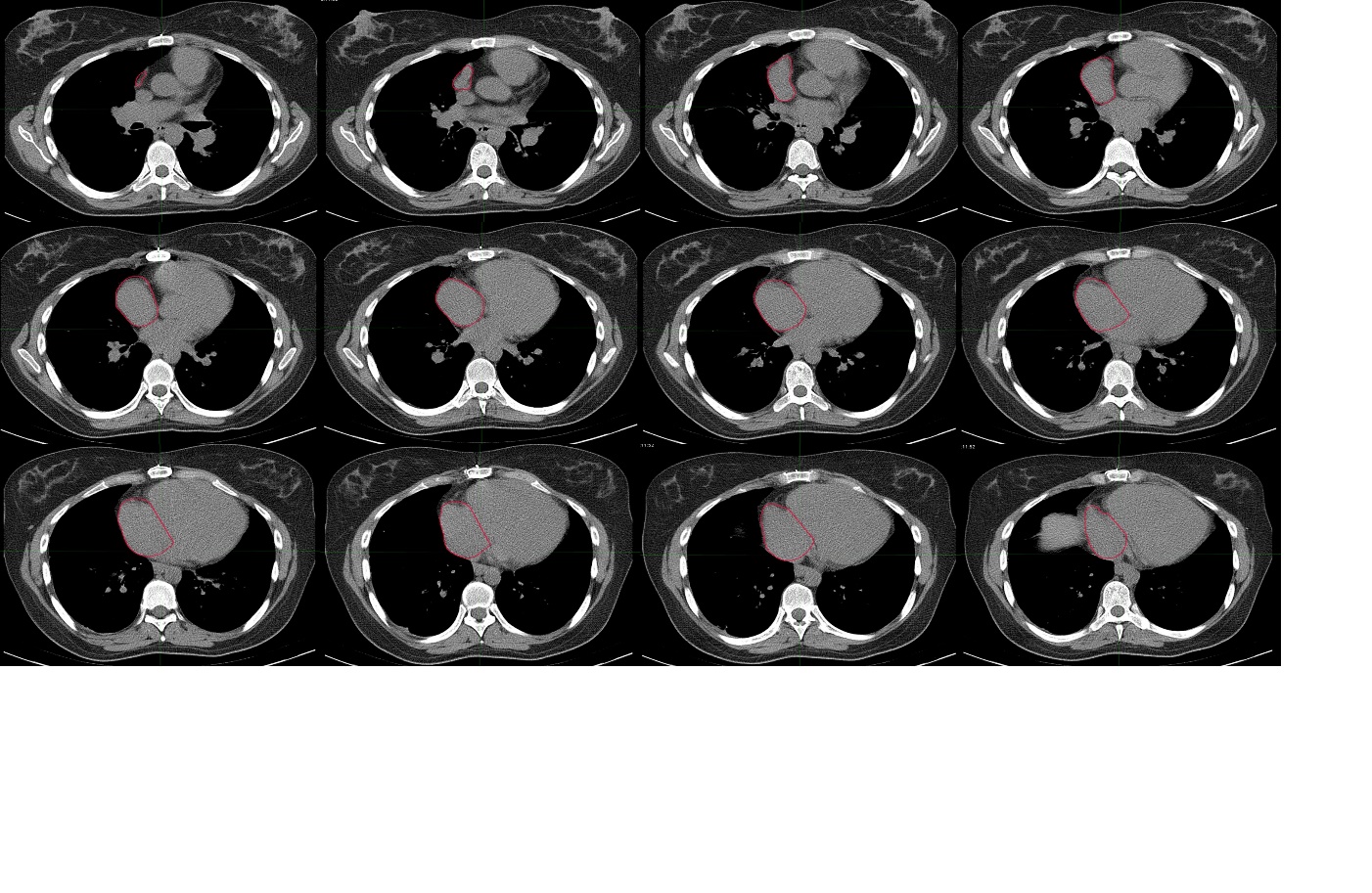


**Supplemental Figure 2.** Right atrium manual annotations.

LA annotation starts from where the structure was visualised. Pulmonary vein and left atrial appendage were excluded. Supplemental Figure 3 presents an example of the LA manual segmentation.


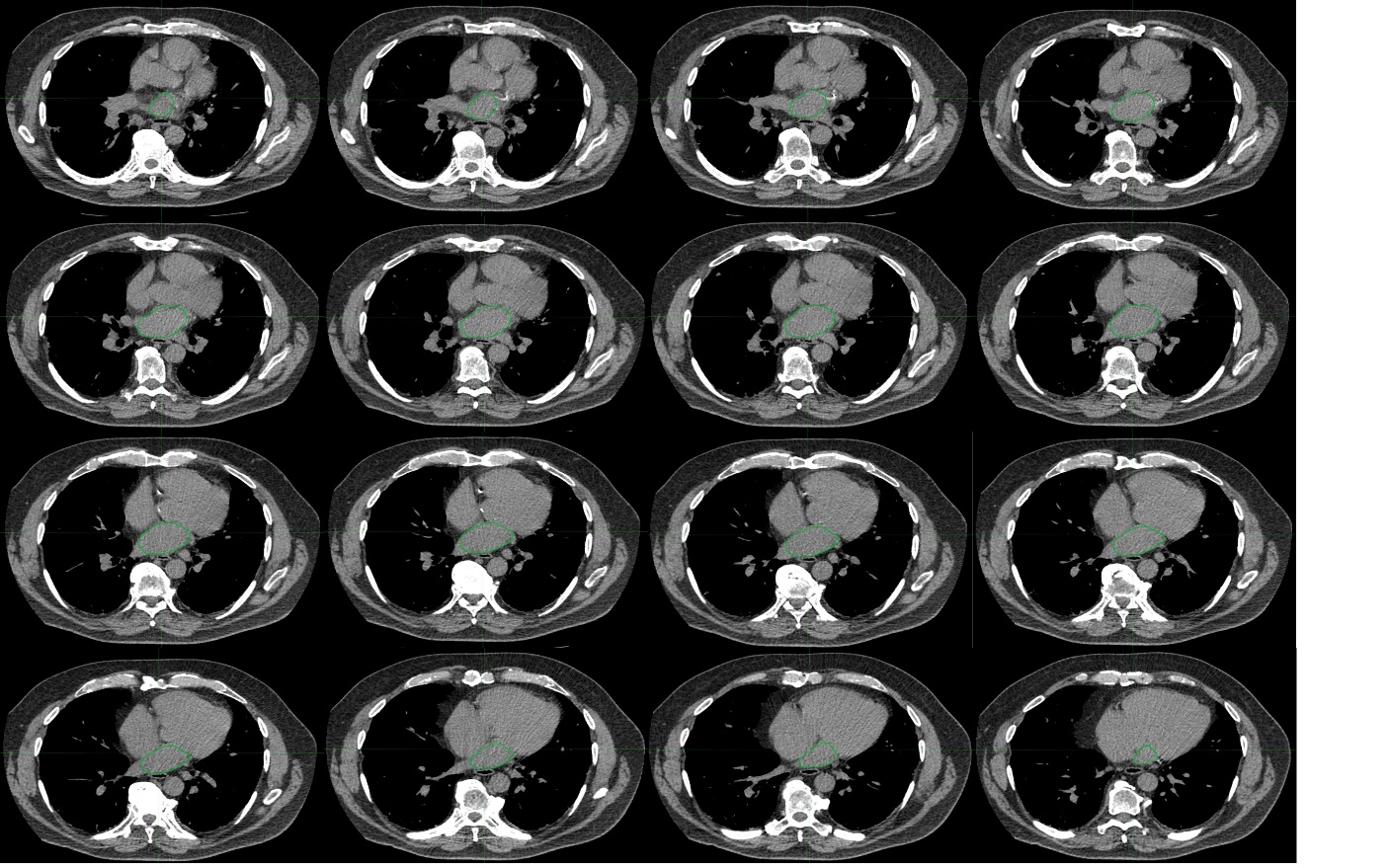


**Supplemental Figure 3.** Left atrium manual annotations.

Ascending aorta annotation starts from the beginning of the aorta until there is an overlap with the ventricles. Supplemental Figure 4 presents an example of the ascending aorta manual segmentation.


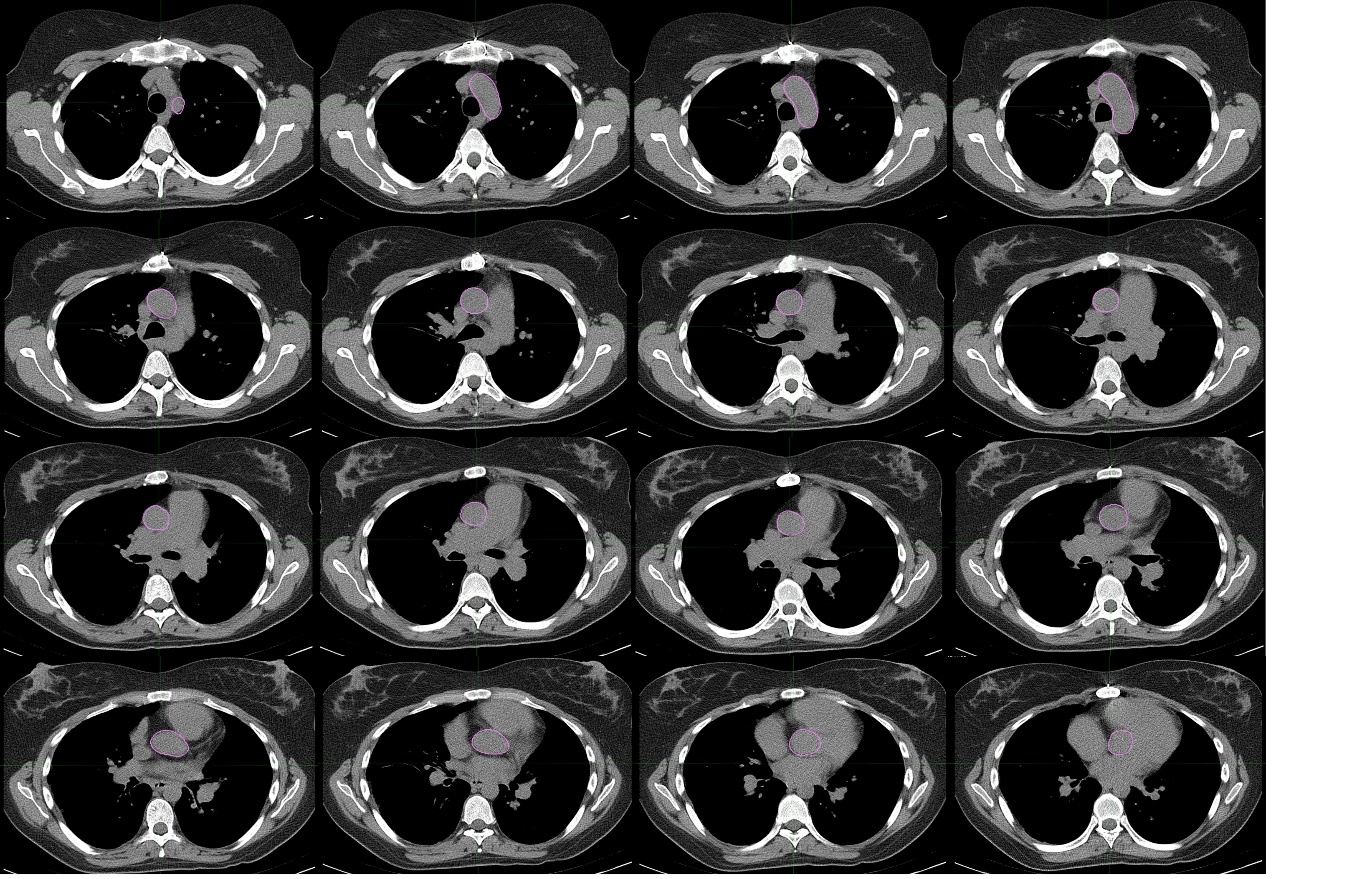


**Supplemental Figure 4.** Ascending aorta manual annotations.

Descending aorta annotation starts at the end of the aortic arch to the abdominal aorta. Supplemental Figure 5 shows an example of the descending aorta manual segmentation.


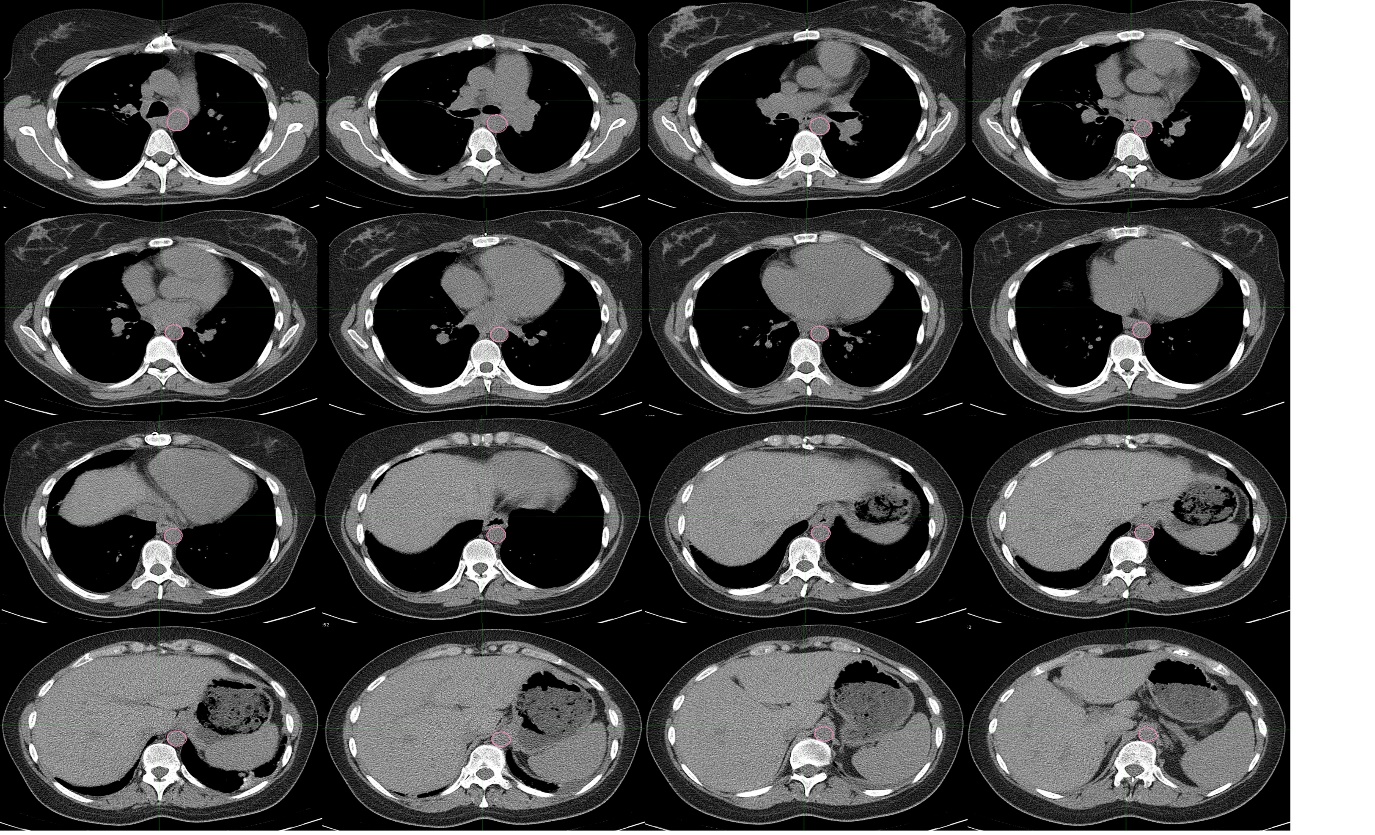


**Supplemental Figure 5.** Descending aorta manual annotations.

PA annotation starts from the base of the right ventricle until there is an overlap with the lungs and airways, including the right and left main arteries, before branching to the segmental level. Supplemental Figure 6 presents an example of the pulmonary artery manual segmentation.


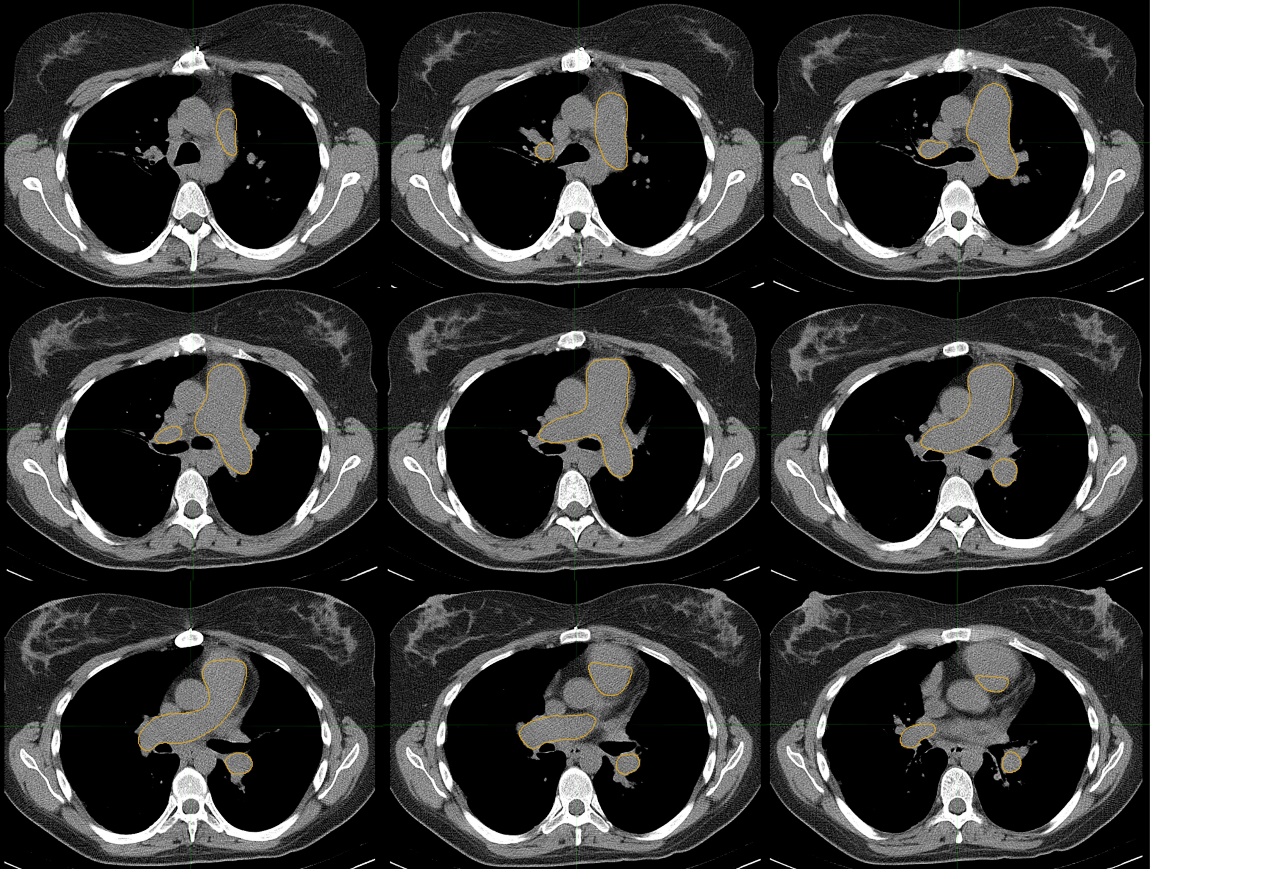


**Supplemental Figure 6.** Pulmonary artery manual annotations.

The trachea and airways were annotated by using the region grow mode tool in MIM software, where the Hounsfield unit can be selected and adjusted to automatically detect the air regions. However, in some cases, the region grow tool failed and the oesophagus had been highlighted due to the similarity of the Hounsfield units to the trachea. Hence, the 2D brush tool was used to correct these cases. The annotation starts from the beginning of the trachea until the bifurcation of the right and left main bronchi. Supplemental Figure 7 presents an example of the trachea and airways manual segmentations.


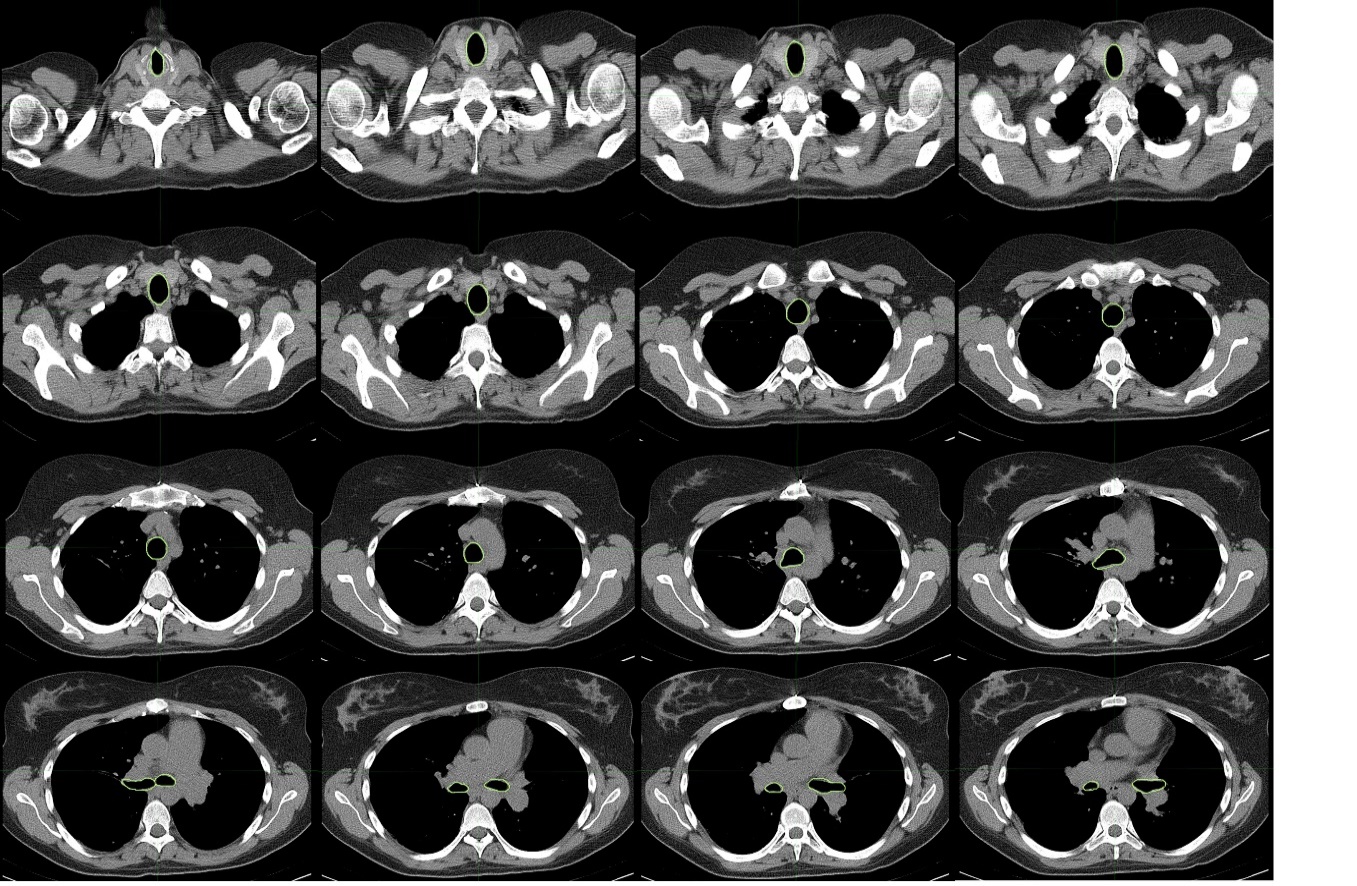


**Supplemental Figure 7.** Trachea and airways manual annotations.

Oesophagus annotation starts from the beginning of the oesophagus at cervical vertebra 6 (C6) until the stomach. Supplemental Figure 8 presents an example of the oesophagus manual segmentation.


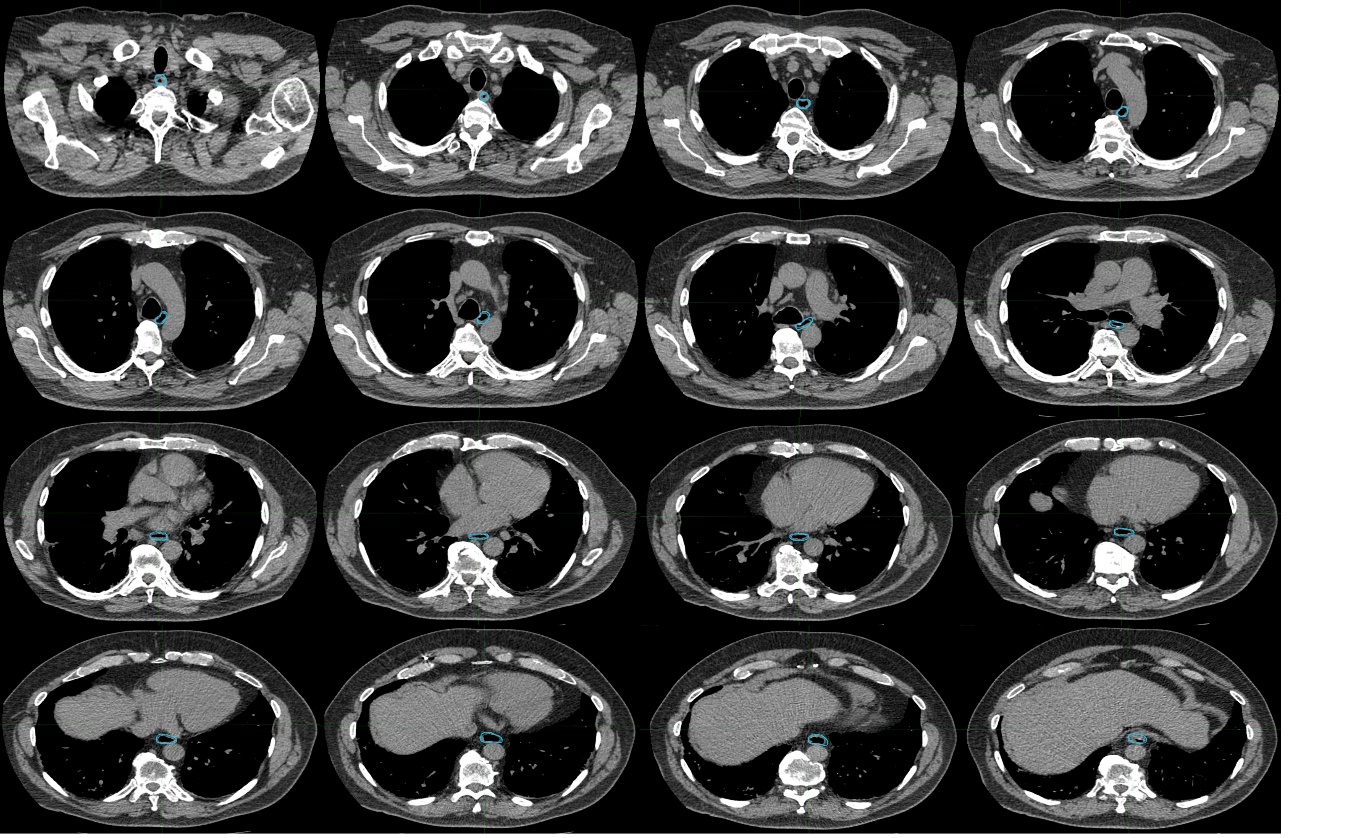


**Supplemental Figure 8.** Oesophagus manual annotations.

SVC annotation starts from the upper confluence point of the brachiocephalic veins until it drains into the superior aspect of the RA. Conversely, the IVC annotation starts from the lower confluence point of the right and left common iliac veins until it drains into the inferior aspect of the RA. Supplemental Figure 9 presents an example of the SVC and IVC manual annotations.


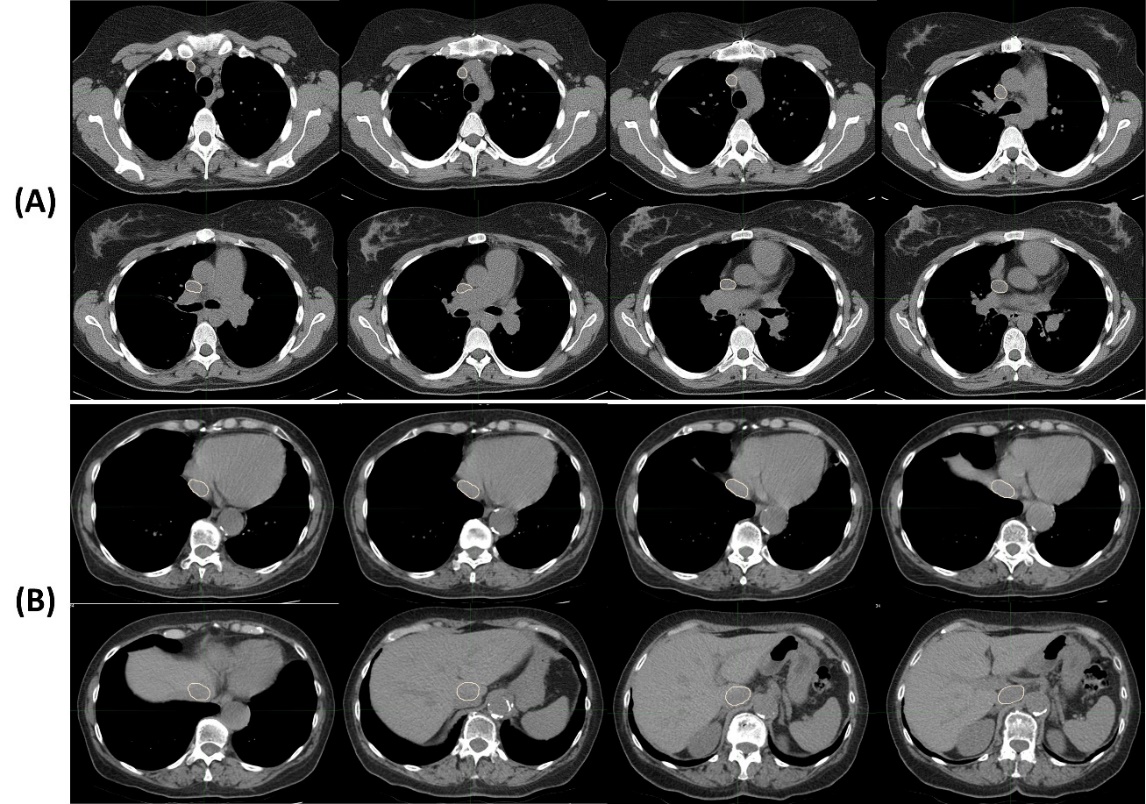


**Supplemental Figure 9. (A)** SVC and **(B)** IVC manual annotations.

Mediastinal fat was annotated by using the 2D brush and pen mode tools in MIM software. Supplemental Figure 10 shows an example of the mediastinal fat manual annotations.


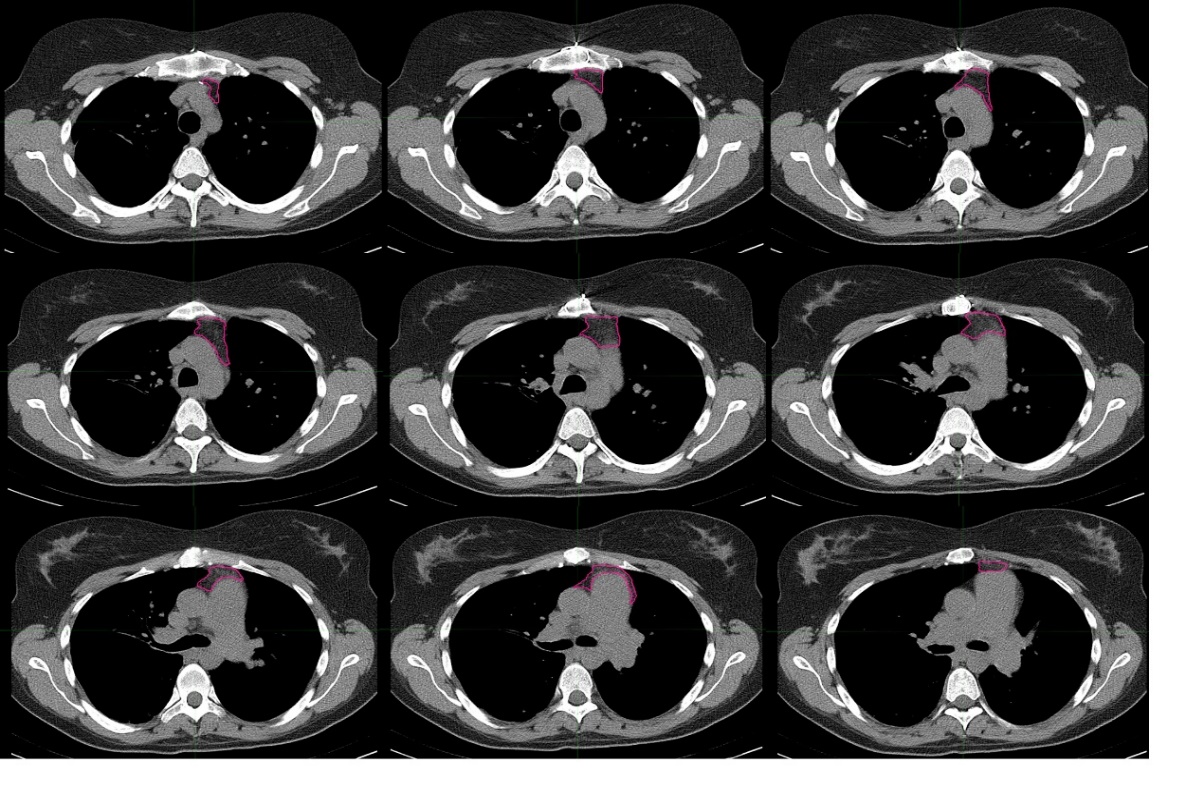


**Supplemental Figure 10.** Mediastinal fat manual annotations.

**
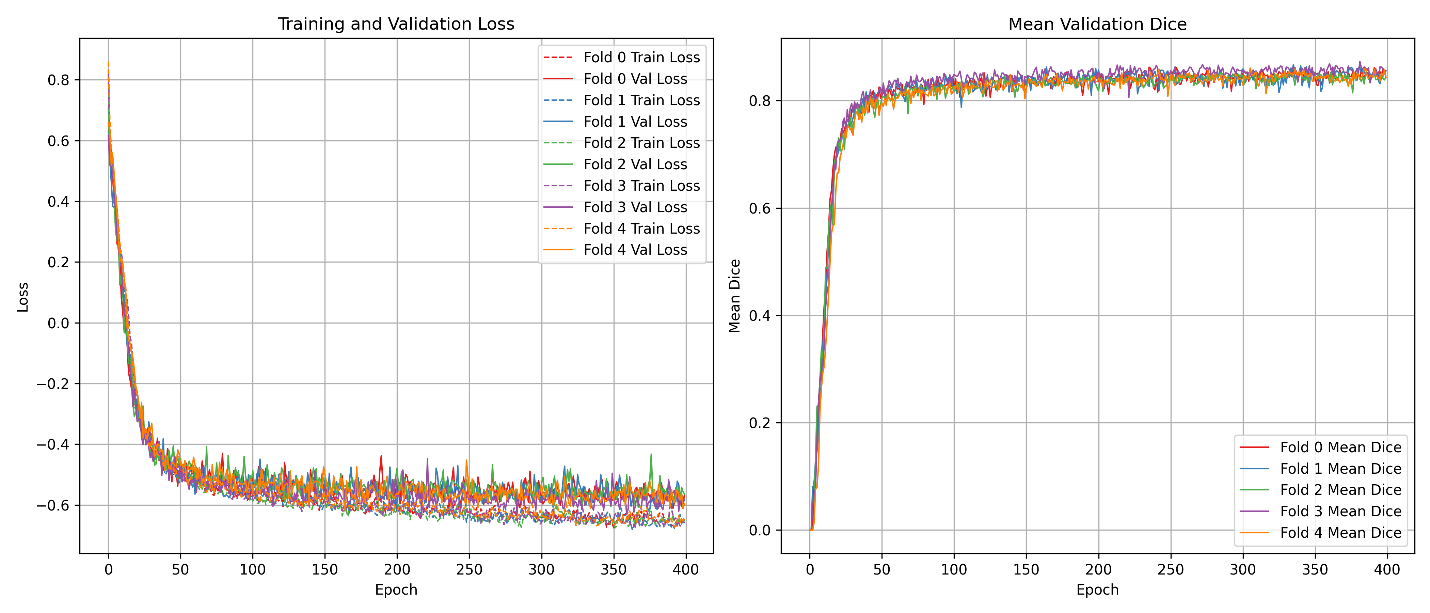
**

**Supplemental Figure 11.** Training and validation loss (left) and mean Dice coefficient (right) across five cross-validation folds, demonstrating stable performance and effective overfitting mitigation.


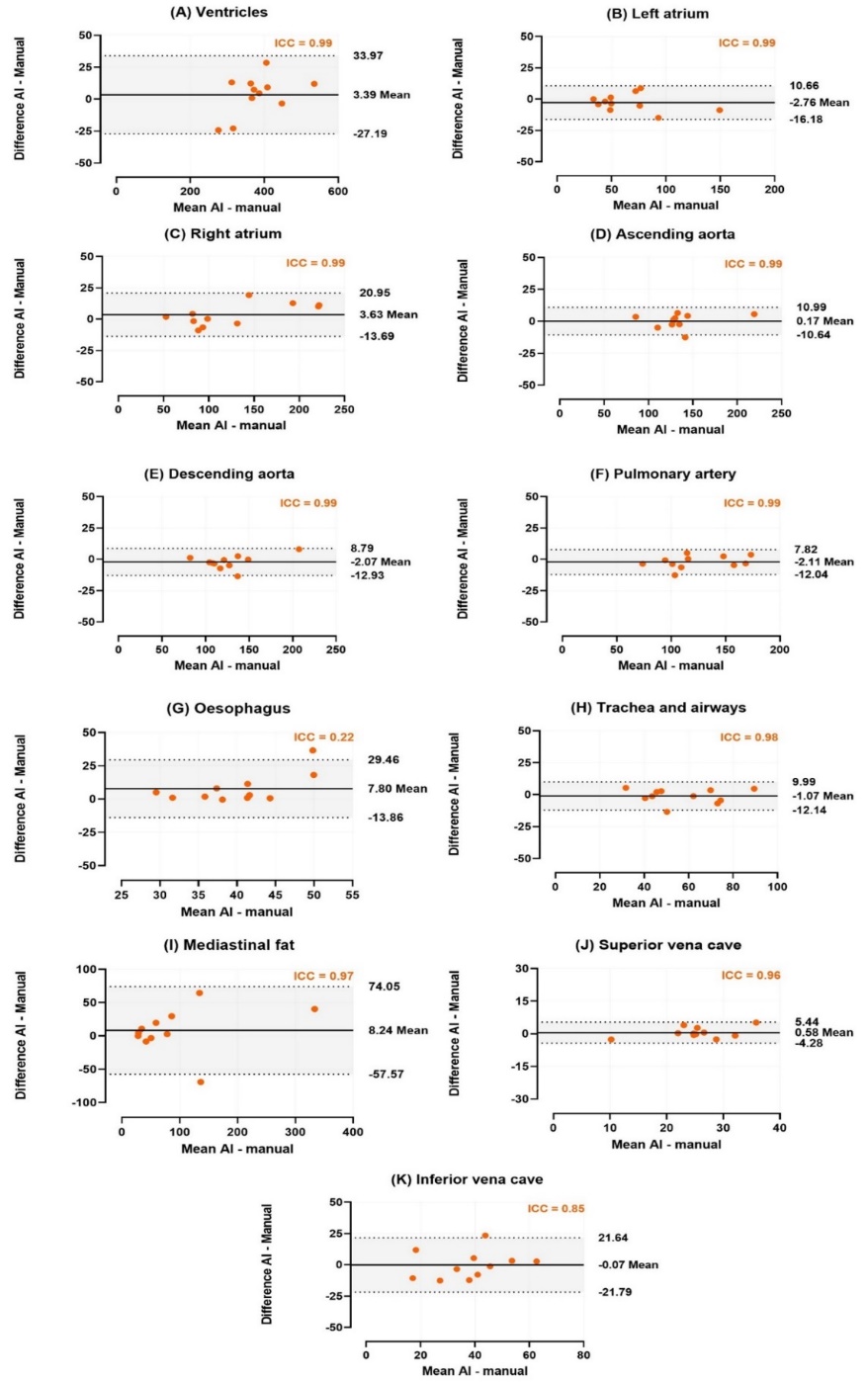


**Supplemental Figure 12.** Bland-Altman plots show intraclass correlations of the segmented cardiothoracic structures in the internal cohort.


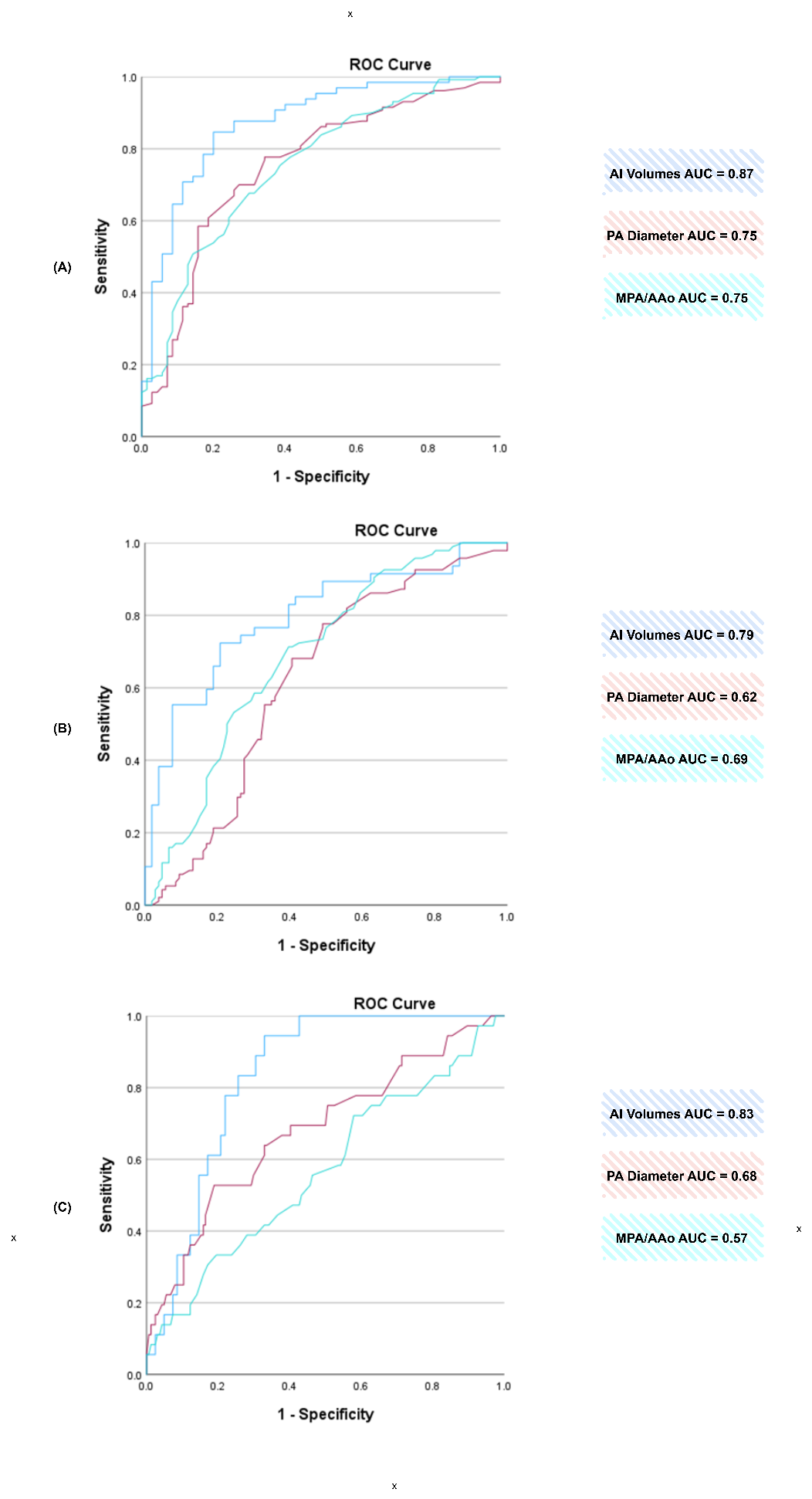


**Supplemental Figure 13.** Exploratory comparison between AI volumetric measurements and MPA diameter and MPA/AAo in prediction of **(A)** PH, **(B)** precapillary PH and **(C)** PH-LHD using the same patients (n=100).


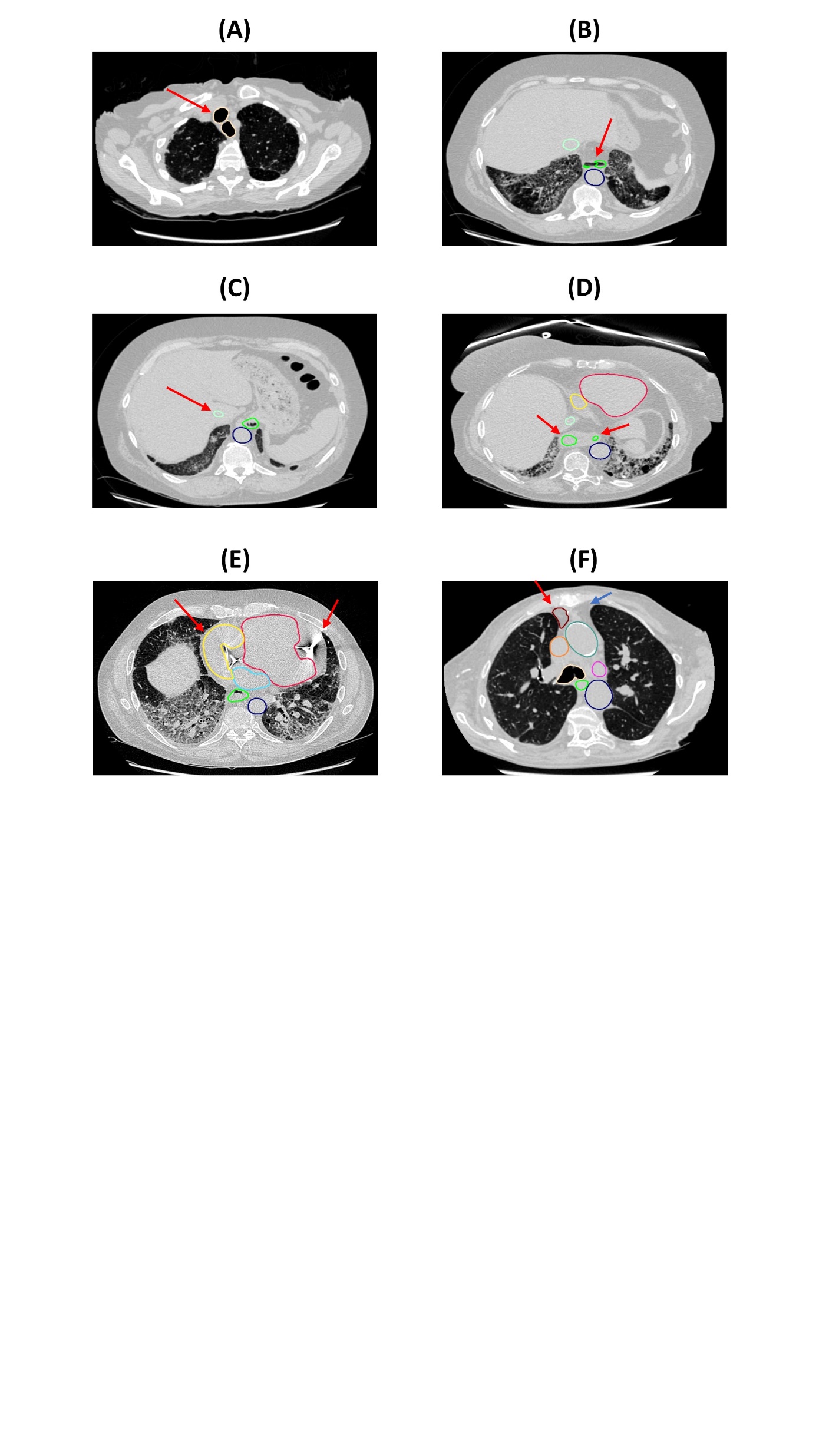


**Supplemental Figure 14.** Examples of failed segmentation cases. **(A)** trachea and oesophagus were combined **(B)** failed oesophagus segmentation **(C)** failed IVC segmentation **(D)** failed oesophagus segmentation and calcifications affecting the segmentation quality of the RA and ventricles **(E)** and mediastinal fat **(F)**.

**Supplemental Tables**

**Supplemental Table 1.** Visual assessment criteria.

| **Scale** | **Description** |
| --- | --- |
| **1: Excellent segmentation** | Highly reliable segmentation. |
| **2: Minor error** | Error in segmentation but considered by the observer to not affect the measurements. |
| **3: Significant error** | Error in segmentation considered by the observer to affect the measurements. |

**Supplemental Table 2.** DSC scores for the tested cohort in the model development stage (n=11).

| **Patient number** | **Ventricles** | **LA** | **RA** | **Ascending aorta** | **PA** | **Descending aorta** | **Oesophagus** | **Airways** | **Fat** | **IVC** | **SVC** |
| --- | --- | --- | --- | --- | --- | --- | --- | --- | --- | --- | --- |
| **1** | 0.87 | 0.72 | 0.86 | 0.91 | 0.64 | 0.92 | 0.55 | 0.78 | 0.82 | 0.67 | 0.87 |
| **2** | 0.95 | 0.92 | 0.91 | 0.95 | 0.93 | 0.96 | 0.73 | 0.83 | 0.83 | 0.79 | 0.92 |
| **3** | 0.96 | 0.87 | 0.93 | 0.97 | 0.90 | 0.94 | 0.82 | 0.93 | 0.77 | 0.78 | 0.91 |
| **4** | 0.97 | 0.93 | 0.85 | 0.96 | 0.94 | 0.95 | 0.80 | 0.92 | 0.69 | 0.55 | 0.88 |
| **5** | 0.95 | 0.87 | 0.90 | 0.96 | 0.93 | 0.96 | 0.82 | 0.93 | 0.64 | 0.64 | 0.84 |
| **6** | 0.93 | 0.90 | 0.92 | 0.95 | 0.92 | 0.95 | 0.89 | 0.93 | 0.75 | 0.85 | 0.89 |
| **7** | 0.96 | 0.89 | 0.92 | 0.93 | 0.92 | 0.96 | 0.85 | 0.93 | 0.84 | 0.55 | 0.87 |
| **8** | 0.94 | 0.85 | 0.92 | 0.94 | 0.86 | 0.95 | 0.76 | 0.91 | 0.71 | 0.64 | 0.78 |
| **9** | 0.96 | 0.88 | 0.90 | 0.92 | 0.95 | 0.92 | 0.78 | 0.90 | 0.71 | 0.89 | 0.86 |
| **10** | 0.95 | 0.89 | 0.91 | 0.96 | 0.92 | 0.96 | 0.81 | 0.94 | 0.71 | 0.87 | 0.89 |
| **11** | 0.97 | 0.90 | 0.89 | 0.95 | 0.95 | 0.95 | 0.83 | 0.89 | 0.83 | 0.84 | 0.90 |

**Supplemental Table 3.** Hausdorff 95% percentile scores for the tested cohort in the model development stage (n=11).

| **Patient number** | **Ventricles** | **LA** | **RA** | **Ascending aorta** | **PA** | **Descending aorta** | **Oesophagus** | **Airways** | **Fat** | **IVC** | **SVC** |
| --- | --- | --- | --- | --- | --- | --- | --- | --- | --- | --- | --- |
| **1** | 3.64 | 4.38 | 3.95 | 1.88 | 7.83 | 2.25 | 28.96 | 10.08 | 2.80 | 6.25 | 1.88 |
| **2** | 0.59 | 1.17 | 1.17 | 0.59 | 0.59 | 0.59 | 1.76 | 1.76 | 1.76 | 2.94 | 1.17 |
| **3** | 0.70 | 0.99 | 0.70 | 0.70 | 0.70 | 0.70 | 2.22 | 0.70 | 5.63 | 2.22 | 0.99 |
| **4** | 0.73 | 0.73 | 3.72 | 0.73 | 0.73 | 0.73 | 1.63 | 0.73 | 80.13 | 19.72 | 0.73 |
| **5** | 3.22 | 3.88 | 3.61 | 1.65 | 2.47 | 1.25 | 2.50 | 2.07 | 27.72 | 18.18 | 3.88 |
| **6** | 0.70 | 0.99 | 0.70 | 0.70 | 0.70 | 0.99 | 0.70 | 0.70 | 13.36 | 1.41 | 1.41 |
| **7** | 0.75 | 1.50 | 0.75 | 0.75 | 0.75 | 0.75 | 1.50 | 0.75 | 2.24 | 30.03 | 0.75 |
| **8** | 0.68 | 1.36 | 0.68 | 0.68 | 2.05 | 0.96 | 10.25 | 0.68 | 6.85 | 8.62 | 1.36 |
| **9** | 0.70 | 0.70 | 0.99 | 0.70 | 0.70 | 0.99 | 4.22 | 2.81 | 25.47 | 1.56 | 2.11 |
| **10** | 0.77 | 0.77 | 1.09 | 0.77 | 0.77 | 0.77 | 6.35 | 0.77 | 270.39 | 1.54 | 0.77 |
| **11** | 0.70 | 0.70 | 1.41 | 0.70 | 0.70 | 0.70 | 2.11 | 1.41 | 4.97 | 2.81 | 0.70 |

**Supplemental Table 4.** Manual volumetric measurements in millilitres (ground truth).

| **Patient number** | **Ventricles** | **LA** | **RA** | **Ascending aorta** | **PA** | **Descending aorta** | **Oesophagus** | **Airways** | **Fat** | **IVC** | **SVC** |
| --- | --- | --- | --- | --- | --- | --- | --- | --- | --- | --- | --- |
| **1** | 264 | 86 | 154 | 135 | 99 | 130 | 68 | 43 | 31 | 21 | 25 |
| **2** | 388 | 145 | 228 | 134 | 116 | 83 | 37 | 34 | 80 | 32 | 27 |
| **3** | 370 | 75 | 226 | 129 | 94 | 121 | 42 | 70 | 28 | 37 | 32 |
| **4** | 367 | 73 | 84 | 125 | 117 | 113 | 32 | 49 | 101 | 24 | 25 |
| **5** | 420 | 44 | 82 | 222 | 167 | 211 | 41 | 72 | 102 | 55 | 25 |
| **6** | 304 | 81 | 84 | 136 | 72 | 103 | 38 | 43 | 49 | 45 | 22 |
| **7** | 541 | 48 | 130 | 108 | 106 | 125 | 45 | 62 | 354 | 32 | 28 |
| **8** | 318 | 33 | 54 | 87 | 97 | 106 | 59 | 39 | 39 | 12 | 9 |
| **9** | 413 | 43 | 99 | 131 | 149 | 108 | 32 | 47 | 69 | 42 | 27 |
| **10** | 446 | 50 | 199 | 129 | 175 | 138 | 47 | 92 | 166 | 64 | 38 |
| **11** | 376 | 36 | 90 | 146 | 156 | 149 | 43 | 72 | 37 | 55 | 25 |

**Supplemental Table 5.** AI volumetric measurements in millilitres.

| **Patient number** | **Ventricles** | **LA** | **RA** | **Ascending aorta** | **PA** | **Descending aorta** | **Oesophagus** | **Airways** | **Fat** | **IVC** | **SVC** |
| --- | --- | --- | --- | --- | --- | --- | --- | --- | --- | --- | --- |
| **1** | 288 | 100 | 135 | 148 | 103 | 144 | 32 | 57 | 26 | 33 | 21 |
| **2** | 384 | 154 | 216 | 136 | 115 | 82 | 35 | 29 | 77 | 35 | 26 |
| **3** | 357 | 69 | 216 | 128 | 95 | 121 | 41 | 76 | 28 | 45 | 33 |
| **4** | 366 | 78 | 93 | 128 | 112 | 120 | 31 | 46 | 71 | 12 | 25 |
| **5** | 391 | 53 | 84 | 216 | 170 | 203 | 33 | 68 | 171 | 32 | 25 |
| **6** | 327 | 72 | 80 | 129 | 76 | 106 | 38 | 44 | 52 | 46 | 22 |
| **7** | 529 | 51 | 133 | 113 | 113 | 130 | 44 | 63 | 313 | 44 | 30 |
| **8** | 305 | 33 | 52 | 84 | 110 | 109 | 41 | 42 | 29 | 22 | 12 |
| **9** | 404 | 45 | 98 | 129 | 147 | 112 | 27 | 45 | 49 | 37 | 24 |
| **10** | 449 | 48 | 186 | 127 | 172 | 136 | 36 | 87 | 102 | 61 | 33 |
| **11** | 368 | 40 | 97 | 142 | 160 | 149 | 40 | 77 | 46 | 52 | 25 |

**Supplemental Table 6.** Comparison of mean values and correlation between two observers for measurements of pulmonary artery (PA), ascending aorta diameter, and PA/Aorta ratio.

|  | **Total included patients (n= 100)** | | |
| --- | --- | --- | --- |
|  | **PA diameter** | **AO diameter** | **MPA/AAo Ratio** |
| **Observer 1** | 33.6 ± 5.8 | 33.2 ± 4.6 | 1.02 ± 0.2 |
| **Observer 2** | 34.7 ± 5.6 | 34.4 ± 4.8 | 1.02 ± 0.2 |
| **Intraclass Correlation Coefficient (ICC)** | 0.86 [0.77 – 0.91] | 0.87 [0.74 – 0.93] | 0.87 [0.81 – 0.91] |

**Supplemental Table 7.** Comparison of PA, ascending aorta diameter, and PA/Aorta ratio between patients with and without PH (p-values included).

|  | | **Patients with PH (n= 65)** | **Patients without PH (n= 35)** | **P-value** |
| --- | --- | --- | --- | --- |
| **Observer 1** | **PA diameter** | 35.0 ± 5.6 | 30.9 ± 5.3 | < 0.001 |
|  | **AO diameter** | 33.0 ± 4.8 | 33.5 ± 4.5 | 0.62 |
|  | **PA/Ao Ratio** | 1.07 ± 0.2 | 0.93 ± 0.1 | < 0.001 |
| **Observer 2** | **PA diameter** | 36.5 ± 5.2 | 31.4 ± 4.8 | < 0.001 |
|  | **AO diameter** | 34.4 ± 4.7 | 34.4 ± 5.0 | 0.76 |
|  | **PA/Ao Ratio** | 1.07 ± 0.1 | 0.92 ± 0.1 | < 0.001 |

**Supplemental Table 8.** Possible reasons for failure.

| **Segmented Structure** | **Possible Failed Reasons** | **Associated Figure** |
| --- | --- | --- |
| **Trachea and airways** | - Due to similar Hounsfield units in the oesophagus. | Supplemental Figure 14A |
| **Oesophagus** | - Image-level issues – such as noise and low quality. - Difficulties in manual annotations of the oesophagus because our cohort included patients with different diseases and degrees of cardiac chamber and great vessel dilatation, which compress the oesophagus. - Small number of cases with a dilated oesophagus in the model’s training and development. | Supplemental Figure 14B  Supplemental Figure 14D |
| **IVC** | - Observers reported that as the number of slices increases and as the slice thickness decreases, the model yields better segmentation quality. | Supplemental Figure 14C |
| **RA, Ventricles, mediastinal fat** | - Calcifications or artefacts can negatively affect the segmentation quality. | Supplemental Figure 14E Supplemental Figure 14F |
